# Household Food Waste: Multivariate Regression and Principal Components Analyses of Awareness and Attitudes among U.S. Consumers

**DOI:** 10.1371/journal.pone.0159250

**Published:** 2016-07-21

**Authors:** Danyi Qi, Brian E. Roe

**Affiliations:** Department of Agricultural, Environmental and Development Economics, Ohio State University, Columbus, OH, United States of America; Utrecht University, NETHERLANDS

## Abstract

We estimate models of consumer food waste awareness and attitudes using responses from a national survey of U.S. residents. Our models are interpreted through the lens of several theories that describe how pro-social behaviors relate to awareness, attitudes and opinions. Our analysis of patterns among respondents’ food waste attitudes yields a model with three principal components: one that represents perceived practical benefits households may lose if food waste were reduced, one that represents the guilt associated with food waste, and one that represents whether households feel they could be doing more to reduce food waste. We find our respondents express significant agreement that some perceived practical benefits are ascribed to throwing away uneaten food, e.g., nearly 70% of respondents agree that throwing away food after the package date has passed reduces the odds of foodborne illness, while nearly 60% agree that some food waste is necessary to ensure meals taste fresh. We identify that these attitudinal responses significantly load onto a single principal component that may represent a key attitudinal construct useful for policy guidance. Further, multivariate regression analysis reveals a significant positive association between the strength of this component and household income, suggesting that higher income households most strongly agree with statements that link throwing away uneaten food to perceived private benefits.

## Introduction

About one-third of the world’s edible food is lost or wasted annually [1], while the challenge to feed the projected world population of 9.3 billion people by the mid-century will require 60% more food than is currently produced [2–5]. The Organization for Economic Co-operation and Development identified reducing food waste as an avenue to increase the availability of food [6], while the Obama administration announced in September of 2015 a first ever food waste reduction goal for the United States of 50% by 2030. While present in the entire post-harvest supply chain, food waste at the retail and consumer levels is particularly prevalent in the United States. In 2010 133 billion pounds of edible food at the retail and consumer levels went uneaten (1,249 calories per person per day) with about two-thirds of this waste attributed to consumers [7]. This represents not only a significant waste of resources, but also substantial negative environmental externalities as 95% of food waste enters U.S. landfills. Food waste is the largest source (35.2 million tons) and the most deleterious component (in terms of greenhouse gas emission) of U.S. municipal solid waste [8–11].

Because so much food waste is attributable to consumers, it is critical to understand consumer awareness, perceptions, opinions, and attitudes that could partly explain the high level of household food waste [12–14], so that potential interventions aimed at obtaining new U.S. goals can be assessed and prioritized. However, no models of food waste awareness, perceptions, opinions and attitudes have been estimated for U.S. consumers. We add to the limited consumer food waste literature by estimating such models using responses from a national survey of U.S. residents. Our models are interpreted through the lens of several theories that describe how pro-social behaviors relate to awareness, attitudes and opinions (e.g., the Theory of Planned Behavior [15], the Norm Activation Model [16], and the Pro-environmental Behavior model [17]). Our analysis of patterns among respondents’ food waste attitudes yields a model with three principal components: one that represents perceived practical benefits households may lose if food waste were reduced, one that represents the guilt associated with food waste, and one that represents whether households feel they could be doing more to reduce food waste. Empirical efforts to assess the relative importance of these key attitudinal responses and principal components and to determine correlations of these key constructs with observable personal and household traits may inform how private and public actions [18–21] could influence behavior.

The existing literature addressing consumer food waste is expanding [22] yet limited and previous work does little to assess the relationships among awareness, attitudes and behaviors. Much of the extant literature summarizes results from regional studies or studies conducted outside the United States [23–29]. However two studies feature similar data or approaches as our work. Neff, Spiker and Truant (2015) [30] report the results from an April 2014 survey, which provides the first national estimates of U.S. consumer awareness, attitudes and behaviors concerning food waste. They report descriptive statistics and pairwise associations between key knowledge, attitude and self-reported behavior measures and several household and personal characteristics, but do not estimate any multivariate regression or principal component models.

The second related study is from Stancu, Haugaard and Lähteenmäki [31] who postulate a consumer food waste model which is estimated with survey data. Their work differs from ours in several ways including their reliance on a sample of Danish consumers and their inclusion of meal planning attitudes and self-reported food waste behaviors, which are elements omitted in our work. However, our survey elicits attitudes that assess the perceived practical benefits of food waste, including attitudes linking food waste to perceived food safety, meal freshness, and time savings, which are factors omitted from [31].

To motivate and frame our work, we draw on previous work that attempts to explain pro-social and pro-environmental behaviors. Our survey work presented below clearly shows that most U.S. consumers perceive actions that reduce food waste as pro-social and pro-environmental behaviors, while previous survey work in the United States [30] show food waste reduction is perceived as pro-social by a majority and pro-environmental by more than 40% of respondents. Motivations for most pro-environmental behaviors include a mixture of self-interested (e.g., energy conservation yielding cost savings) and pro-social (e.g., energy conservation preserving resources for others and reducing pollution) motives [17]. Analyses of behaviors with a mix of possible motivations often build from theories such as the Theory of Planned Behavior (TPB [15]), the Norm-Activation Model (NAM [16]) and models that integrate and expand upon aspects of both TPB and NAM such as the Stancu, Haugaard and Lähteenmäki model of food waste behavior [31] and Bamberg and Moser’s pro-environmental behavior model [17].

Our conceptual model features three key constructs that arise from our empirical investigation and that are motivated from the theoretical traditions discussed above. The first construct is dubbed Practical Benefits of Food Waste; it draws on the self-interest side of the theoretical literature and will represent the private perceived benefits households accrue by wasting food. The second construct is dubbed Food Waste Guilt and it draws upon constructs of norms and related concepts of guilt. Our third and final construct is dubbed Food Waste Reduction Potential, which is motivated by the concept of Perceived Behavioral Control (PBC), a key antecedent of behavioral intentions in the TPB.

The remainder of the article is organized as follows. First we discuss our survey instrument and methods. We then provide descriptive statistics and multivariate analysis of key food waste awareness and attitudinal questions. We then present the estimated principal components model and related multivariate analyses, followed by a discussion of the results, implications and limitations of the study.

## Materials and Methods

### Survey Development

To enhance comparability with previous research, awareness and attitudinal questions were drafted to measure similar concepts explored by food waste surveys known to the authors at the time of survey development (e.g., WRAP 2014 [28]). Prospective language was shared with professional survey developers at the research firm administering the survey, and then revised to accommodate input received.

A single awareness question (“In the last 12 months, have you read, seen or heard anything about the amount of food that is wasted or about ways to reduce the amount of food that is wasted?”) was developed featuring a simple yes/no response (yes coded 1, no coded 0). Respondents were coded as ‘unsure’ only after being read the question twice and volunteering an inability to choose a response. In regression analyses uncertain responses were coded as 0.5. If uncertain responses were instead dropped from the analysis, no statistically significant coefficients change sign, fewer than 7.2% of estimated coefficients change significance status, and fewer than 7.2% of all coefficients change signs.

**Fig 1:**
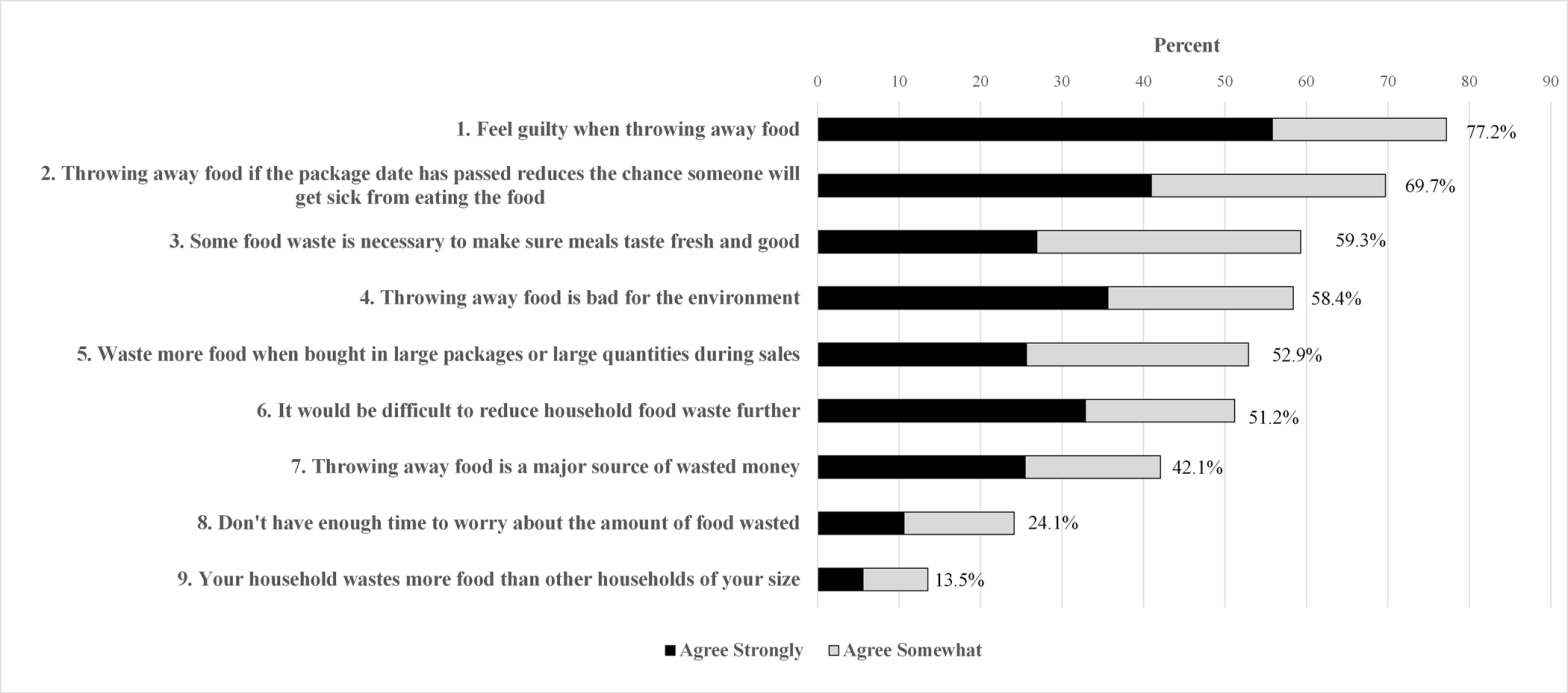
Agreement with statements related to food waste.

The generation of food waste is a result of multiple behaviors that relate to different aspects of food purchasing, preparation, consumption and post-meal behaviors [32]. Based on literature reviewed above and based on questions implemented in previous surveys [30, 32], a slate of nine statements relating food waste to these aspects were presented in the survey (see Fig 1 for paraphrased statements and S2 File for exact wording). Statements 2, 3 and 8 focus on private, practical benefits that a household may perceive to accrue from throwing away uneaten food such as reduced odds of foodborne illness, improved meal freshness, and time savings; each loads onto the first construct. Statements 1, 4 and 7 focus on potential sources of guilt or norm comparison thought to load onto the second construct. Statements 5, 6, and 9 focus on perceived behavioral control and benchmarking concepts thought to load onto our third construct.

Respondents expressed agreement or disagreement on a four point Likert scale (agree strongly, agree somewhat, disagree somewhat, disagree strongly). In all analyses, the responses to these questions were recoded from 1 to 4 with larger numbers indicating stronger agreement with the given statement. Respondents were coded as ‘unsure’ only after being read the question twice and volunteering an inability to choose a response. Uncertain responses occur in 1.8% of responses across these questions and never exceed 4.1% of the total weighted sample for any question. Hence, in all analyses of attitudinal questions uncertain responses were dropped. Four other questions concerning food waste were asked as part of this survey, but are not the focus of this analysis and so are excluded from this article.

### Sampling and Implementation

The survey was administered by SSRS as part of a weekly national dual-frame telephone omnibus study conducted by the firm via computer-assisted interviewing. The sample is designed to be representative of the United States (including Hawaii and Alaska) and features a fully-replicated, stratified, single-stage random-digit-dialing sample of landline telephone households, and randomly generated cell phone numbers. About 3 to 4 percent of interviews are conducted in Spanish to facilitate proper representation of the Hispanic population.

The response rate reported by SSRS is approximately 8%, which is common for large national omnibus surveys that are conducted on a weekly basis and do not allow for follow ups beyond the week the survey is launched. Recruitment scripts for the survey do not mention food waste or the other focal topics featured on this multi-topic survey, which may reduce the tendency for those motivated by topics such as food waste to volunteer for the survey and, hence, may mitigate self-selection bias. The vendor made six attempts to contact each sampling unit and used Spanish-speaking interviewers once a household is identified as Spanish speaking. We note that Neff, Spiker and Truant report a response rate of 51%, though this figure is the response rate among online panel members previously recruited to be members of the online panel, and no information is provided about the percent of recruits to online panel membership that become panel members. Hence, direct comparisons between response rates for the two studies are complicated by the different survey sampling methodologies employed. Unless otherwise noted, analyses are conducted with weights designed to create nationally representative and projectable estimates of the adult population 18 years and older on age-by-gender, race and ethnicity, census region, population density of county of residence, and education.

In addition to our slate of questions concerning food waste, each respondent answers questions posed by other clients contracting with SSRS during the week the interviews were conducted. The order of our group of questions is randomized with those from other contributors to the survey, whose identity and questions are unknown to the authors. Each survey ends with a broad array of questions focused on personal and household characteristics of the respondent.

Telephone interviewers were personally briefed and trained on our slate of questions before conducting the interviews. Mock interviews were conducted to ensure that interviewers followed all procedures correctly and consistently. In addition, throughout the week of administration, SSRS field personnel and project directors continually monitored the interviewers to ensure that diction and probes are consistent across interviewers.

### Analysis

Analyses were conducted in SAS 9.3 and Stata 13.1. To understand common patterns among attitudinal responses we extract principal components (SAS 9.3, Proc Factor). Prior communality estimates are set to one and components with an eigenvalue less than one are dropped from the analysis. A varimax variable rotation is used to arrive at the principle components presented in the results section. Significant loadings onto a principle component are defined as those with a loading greater than 0.40 in absolute value.

In order to refine our understanding of the associations between respondents’ awareness and attitudes and respondents’ personal and household characteristics, we conduct multivariate regression analyses (Stata 13.1). An ordered logit regression was used for the awareness response and to model agreement with individual statements listed in Fig 1. Ordinary least squares regression was used to model associations between characteristics and principal components extracted from the attitudinal responses. Control variables, which are the same across all the regression models, include household demographics covered in existing food waste literature, such as education, income, age, gender, marital status, household size and composition, employment status, urbanicity and region [27, 30]. Other variables are also included that feasibly affect food waste awareness and attitudes. Home ownership is included as it is strongly correlated with U.S. residential size: owned residences feature greater total square footage (1800 vs. 974 median square feet), per person square footage (800 vs 500 median square feet), and lot size (0.31 versus 0.18 median acres[33, 34]). Residence and lot size may influence whether respondents can invest in items associated with food storage, leftover storage, and food waste handling such as larger refrigerators, stand-alone freezers, greater pantry space, or outdoor composting sites. Given attitudinal questions exploring feelings of guilt, we include religious affiliation as a possible control variable. We include race as a possible control for different food preferences and different food preparation and dining habits. We use a 5% level to determine statistical significance and denote the *p-*value of variables in all tables.

### Ethics Statement

This study and its consent procedure were reviewed and approved by the Ohio State University Institutional Review Board. Survey respondents contributing to this study provided verbal consent to participate in the questions asked while survey recruits that did not consent were not asked any study questions.

## Results

We first report descriptive statistics for all variables and compare these to recent U.S. survey work before discussing results from multivariate models of each individual awareness and attitudinal response. We then present the principal components analysis and accompanying multivariate analysis of associations with personal and household characteristics.

Key personal and household characteristics of the unweighted sample are listed in Table 1 and compared to national figures. While the unweighted figures align well with national averages on several characteristics, survey weights are applied to all subsequent analyses to further refine the representativeness of the sample.

**Table 1:** Respondent Demographics, Unweighted^+^.

		**Survey (%)**	**U.S. (%)**
**Gender**			
	Male	48	49
	Female	52	51
**Age**			
	18–29	16.2	22^a^
	30–49	25.6	34^a^
	50–64	27.2	26^a^
	65^**+**^.	30.6	19^a^
**Education**			
	Less than high school	8.8	12
	High school	34.1	30
	Some college	23.5^b^	30
	College graduate	18.6	19
	Graduate school or more	15.0	9
**Race**			
	White, Non-Hispanic	73.2	62
	Black, Non-Hispanic	10.6	12
	Other, Non-Hispanic	5.5	8
	Hispanic	10.8	17
**Household Income**^**c**^			
	Less than $50,000	57.9	48
	$50,000-$99,999	25.6	41.9
	More than $100,000	16.5	10.1
**Household Size**		2.7	2.6
**% Married**		46.8	52
**% with Health Insurance**		86.4	86.6
**Live with child/ren<18**		29.6	43

^+^Due to rounding, some categories do not sum to 100 percent.

^a^ Percentage is based on the population over 18-year-old, not total population.

^b^ Some college includes people who have some college credits but failed to graduate, and people with degrees from technical school/other which account for 1.6% of the sample.

^c^ Household Income figures represent percentages of those who provided information for this question. 63 of the 500 respondents refused to answer this question.

Source for US data: United States Census Bureau.

### Awareness

A modest majority of the weighted sample (53%) answered ‘yes’ when asked “In the last 12 months, have you read, seen or heard anything about the amount of food that is wasted or about ways to reduce the amount of food that is wasted?” Of the remaining respondents, 39% responded ‘No’ with the remaining 8% uncertain. Neff, Spiker and Truant (2015) asked their respondents a similar question: “In the past year, have you seen or heard anything in the news, social media, or elsewhere about the issue of food that is thrown out or otherwise not eaten by humans? (Sometimes referred to as ‘wasted food’).” 42% answered ‘yes’ to this question, which is 11 percentage points lower than our sample’s awareness response, a difference that is statistically significant (*z* = 4.05, *p* < 0.001).

### Attitudes and Opinions

Respondents’ agreement with the slate of nine food waste attitudinal statements is listed in Fig 1. The order of the statements as read to respondents was randomly assigned to mitigate possible order effects. Uncertain responses only average 1.8% across these questions and never exceed 4.1% of the total weighted sample for any question, which accounts for a much smaller percentage of the sample than 8% uncertain respondents in awareness question. Hence, to simplify exposition, uncertain responses are dropped, and percentages reported in this section represent the percent of respondents able to articulate a response to each statement.

Respondents express the highest degree of agreement with the statement linking guilt to throwing away food, with more than three quarters agreeing either strongly or somewhat. The next strongest agreement is with a statement suggesting that throwing away food if the food package’s date has passed helps reduce the odds of foodborne illness. More than two-thirds of respondents (69.7%) express agreement despite increased press coverage of scientific literature suggesting that label dates are not a good proxy for foodborne illness threats [35, 36].

The next strongest agreement is with a statement that some food waste is necessary to ensure meal freshness and quality (59.3%) followed by a statement that throwing away food is bad for the environment (58.4%). Respondents are about equally split between agreeing and disagreeing with statements that food waste is exacerbated by bulk and sale purchases (52.9%) and that it would be difficult for their household to reduce food waste further (51.2%).

A minority agrees that changing household food waste levels would induce significant changes in money or time costs, with 42.1% agreeing food waste is a major source of wasted money and 24.1% agreeing that they do not have enough time to worry about food waste. Only 13.6% agree that their households waste more food than other households of their size.

These results align with the results presented by Neff, Spiker and Truant on several fronts. 65% of Neff, Spiker and Truant’s sample agree that they worry about food poisoning when making food discard decisions, which aligns closely with the 69.7% in our sample that agreed that food waste can reduce the risks of foodborne illness. At the 5% level we fail to reject equality of the proportion of respondents expressing agreement (agree strongly or agree somewhat) on this topic between our survey and the Neff, Spiker and Truant survey (*p =* 0.055, *z* = 1.92). Similar percentages of respondents also agree that food waste is important for ensuring meal quality and freshness (60% for Neff, Spiker and Truant, 59.3% for our sample, *z* = 0.16, *p* = 0.87). The two samples contrast on responses to questions linking food waste to financial waste in respondent households, though the question wording between the surveys was not as closely aligned. For the Neff, Spiker and Truant sample, 63% disagrees with the statement “I don’t think the amount of food I throw away costs me much money” (note the double negative) while 42.1% of our sample agrees that “Throwing away food is a major source of wasted money in your household,” *z* = 7.26, *p* <0.001). A minority of both samples agree with statements linking a lack of time to the amount of food they waste, though the level of agreement is higher in our sample with the difference being statistically significant (15% for Neff, Spiker and Truant, 24.1% for our sample, *z* = 4.08, *p* < 0.001). Finally, few in either sample viewed their own level of food waste to be above average (73% of the Neff, Spiker and Truant sample say they waste less than average, while 86.5% of our sample disagree that they waste more than similarly sized households, *z* = 5.75, *p* < 0.001).

The wording of questions that assess guilt and environmental motivations about household food waste is less comparable between the Neff, Spiker and Truant study and ours. However, a similar pattern emerges where general guilt towards food waste appears to dominate specific concerns about the environment in both studies. For example, in Neff, Spiker and Truant, those reporting that ‘guilt about waste in general’ as an important motivation for reducing food waste is around 60%, while only about 40% cite greenhouse gases, energy and water as an important motivation; that is, general guilt sentiments exceed environmental concerns by about 20 percentage points. In our sample, there is also about a 20 percentage point gap between those agreeing that they feel guilty about food waste (77.2%) and those agreeing that throwing away food is bad for the environment (58.4%).

### Associations with Personal and Household Characteristics

Results from an ordered logit regression model of food waste awareness are reported in Table 2.

**Table 2:** Food waste awareness regression model.

*In the last 12 months*, *have you read*, *seen or heard anything about the amount of food that is wasted or about ways to reduce the amount of food that is wasted*?		Coef.	p-value	Odds Ratio
**Education**	<High school (Omitted)	*Joint p = 0*.*135*		
	High School	0.891	0.055	2.438
	Some College	1.205*	0.014	3.337
	Graduate College	0.866	0.102	2.377
	Graduate School	1.345*	0.022	3.839
**Employment**	Full-time (Omitted)	*Joint p = 0*.006*		
	Part-time	1.191*	0.001	3.289
	Retired	0.925*	0.023	2.521
	Homemaker	0.971	0.118	2.640
	Student	-0.390	0.575	0.677
	Temporarily unemployed	-0.444	0.407	0.642
	Disabled/ handicapped/ other not employed	0.674	0.220	1.962
**# Adult Females in HH**		-0.620*	0.019	0.538
**# Kids in HH**		-0.498*	0.019	0.608
**HH Size**		0.377	0.055	1.458
**Income**	<$50,000 (Omitted)	*Joint p = 0*.*476*		
	$50,000-$99,999	-0.233	0.442	0.793
	More than $100,000	-0.036	0.932	0.964
	Refused/Missing	-0.684	0.148	0.505
**Metro Area**	Center City (Omitted)	*Joint p = 0*.*430*		
	Center City County	-0.303	0.397	0.739
	Suburban	-0.414	0.236	0.661
	Non-Center City	-0.696	0.272	0.499
	Non-Metro	-0.569	0.086	0.566
**Age**	18–29 (Omitted)	*Joint p = 0*.*139*		
	30–49	-0.208	0.628	0.812
	50–64	0.347	0.456	1.414
	65+	-0.389	0.506	0.677
**Homeowner**		0.452	0.108	1.571
**Male**		0.278	0.306	1.321
**Race**	White, non-Hispanic (Omitted)	*Joint p = 0*.*774*		
	Black, non-Hispanic	-0.192	0.651	0.825
	Hispanic	0.277	0.510	1.319
	Other race	0.354	0.557	1.425
**Region**	North East (Omitted)	*Joint p = 0*.*362*		
	North Central	-0.276	0.494	0.759
	South	-0.116	0.75	0.890
	West	0.353	0.389	1.423
**Marital Status**	Single, never married (Omitted)	*Joint p = 0*.*532*		
	Single, live with a partner	0.215	0.679	1.240
	Married	-0.322	0.423	0.725
	Separated	0.537	0.468	1.711
	Widowed	0.022	0.968	1.022
	Divorced	0.239	0.61	1.270
**Religion**	1 (Omitted)	*Joint p = 0*.*499*		
	2	-0.168	0.592	0.845
	3	1.100	0.220	3.004
	4	-1.099	0.134	0.333
	5	-0.003	0.997	0.997
	6	-0.189	0.594	0.828

* p<5%.

Religious categories are as follows: 1 (omitted) includes Baptist, Christian, Church of Christ, Church of God, Evangelical, Holiness, Non-denominational or Independent, Pentecostal; 2 includes Catholic, Congregational or UCC, Episcopalian or Anglican, Lutheran, Methodist, Orthodox, Presbyterian, Unitarian/Universalist, Protestant, 3 includes Buddhist, Hindu, Muslim/Islam; 4 includes Jehovah’s witness, Mormon, Seventh-Day Adventist; 5 includes Jewish; 6 includes Atheist, Agnostic, Other, Nothing in particular, Don’t know, Didn’t report.

While Neff, Spiker and Truant do not report tests results for association between awareness and household demographics, they report two significant associations between food waste knowledge (which we do not ask) and demographics. Specifically, they find those 65 years of age or older express significantly greater knowledge of food waste than those younger than 65, while we find no significant differences in reported awareness across age groups in our regression results. Also, Neff, Spiker and Truant find that households with children under 18 years of age have significantly less knowledge than other households, where we find awareness to be significantly negatively associated with the number of children in a household. We find the employment status variables are jointly significant, with part-time and retired respondents revealing significantly higher awareness than those employed full time. This may reflect that those employed full time have less time or focus available to generate awareness of this issue. While the education variables are not jointly significant, the results do reveal that the omitted group (those without a high school degree) is significantly associated with less awareness than those who have received more formal education.

Multivariate ordinal regression analyses of the association between the attitudinal and opinion statements and respondent characteristics reveal several statistically significant results (see Tables A-I in S1 File). For example there is a statistically significant association between gender and agreement with the statement that the respondent feels guilty when throwing away food, with women more likely to agree with this statement (Table A in S1 File). Likewise, those who reveal awareness of the food waste topic are also more likely to agree with this statement (Table A in S1 File). A respondent’s education is associated with agreement with the statement linking food waste to environmental harm (Table D in S1 File) with those who obtain higher levels of formal education expressing stronger agreement. Those with more formal education are also more likely to agree with the statement linking bulk purchases to increased household food waste (Table E in S1 File). Employment status is jointly significantly associated with a respondent’s agreement with the statement saying the respondent doesn’t have enough time to worry about food waste (Table H in S1 File) with those who are temporarily unemployed, disabled, handicapped, and other not employed featuring the strongest disagreement (or in other words, the strongest agreement that they have sufficient time to worry about reducing household food waste). Finally, there is a jointly significant association between a respondent’s region and a respondent’s agreement that their household tends to waste more food than other similarly sized households (Table I in S1 File) with those from the South and West expressing the least agreement.

### Principal Components Analysis

**Fig 2:**
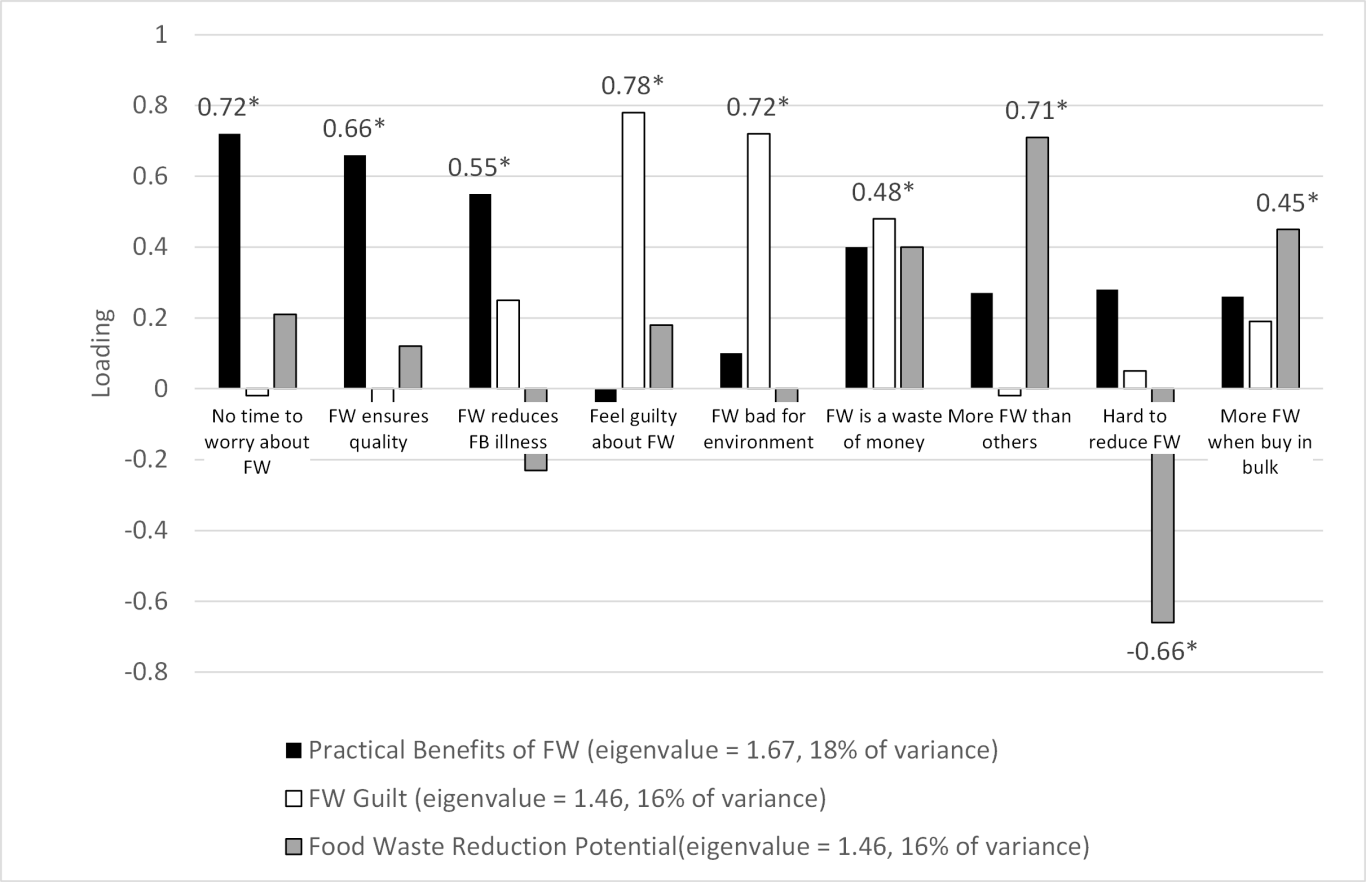
Rotated principal component loadings. No differences in the sign or significance of loadings are found between the results obtained from a varimax and an oblimin rotation. * Denotes loadings greater than 0.40, the chosen threshold for significant loadings. a—FW–food waste. b—FB–foodborne. c—The value for the fourth largest eigenvalue was 0.93, which is less than the threshold of 1.0 and was omitted from further analysis.

Results from our principal components analysis (Fig 2) yields three component where one component (black bars) represents perceived practical benefits households may lose if food waste were reduced, another (white bars) represents the guilt associated with food waste, and the third (grey bars) represents whether households feel they could be doing more to reduce food waste. No other estimated component featured a large enough eigenvalue to be retained. Fig 2 reports the loadings of each attitudinal variable on the three retained, rotated principal components with the eigenvalues of the retained principal components listed in the figure’s legend. The three retained components represent about 50% of response variance and each component captures about the same percent of overall variance (16–18%).

The first principal component features three significant loadings related to agreement with the statement that there isn’t enough time to worry about food waste (0.72), that food waste is necessary to ensure meal freshness and quality (0.66) and that throwing away food whose package date has passed can reduce the chances of foodborne illness (0.55). The ‘Practical Benefits of Food Waste’ component increases as they perceive that wasting food can deliver practical household benefits (more time, improved meal quality/freshness, less chance of foodborne illness). This component aligns with constructs representing the perceived personal benefits for wasting food and costs associated with reductions in food waste. According to the TPB, the balance of such perceptions helps generate attitudes toward the behavior that influences behavioral intentions and, potentially actual behavior.

Wasting food may generate several private benefits for a household. Households may perceive that throwing out uneaten food will minimize health risks from foodborne illness or enhance meal quality by avoiding the use of less fresh food (e.g., leftovers or ingredients with limited remaining shelf life). Respondents may generate food waste as a result of having saved time earlier in the meal planning and preparation stages (e.g., the main shopper did not spend the time needed to carefully plan previous food shopping trips or to think through the storage and subsequent preparation of leftovers). We note this construct is absent in Stancu, Haugaard and Lähteenmäki’s [27] model of consumer food waste.

The second retained component also features three significant positive loadings. The largest loading is from the variable in which respondents agree that they feel guilty for throwing away food (0.78), followed by agreement with the sentiment that throwing away food is bad for the environment (0.72) and that throwing away food is a major source of wasted household money (0.48). This ‘Food Waste Guilt’ component overlaps with norm constructs from several theories mentioned earlier. The positive loading on the statement linking guilt to throwing away food suggests such sentiments arise from the respondents perceiving food waste as a deviation from a norm against wasting food. The positive loading on the statement linking food waste to environmental harm may come from those who view protecting the environment as a norm. Finally, the positive loading on the statement identifying food waste as a source of wasted household money may stem from those who view household financial waste as a deviation from norms of household financial prudence. However, in each case, our instrument cannot distinguish whether the norm potentially invoked is a social or a moral norm.

Social norms are postulated to influence behavioral intentions in the TPB while the NAM postulates moral norms as a direct influence on behavior. Bamberg and Moser’s pro-environmental behavior model [17] postulates that social and moral norms both contribute to behavior, though their empirical application reveals a direct statistical linkage between moral norms and behavioral intentions and only an indirect (mitigating) statistical linkage for social norms via attitude formation. Throwing away food could feasibly activate norms on several fronts including environmental, financial or household management. Stancu, Haugaard and Lähteenmäki [31] feature a moral norm construct with loadings from two statements focused on guilt, though their moral norm construct has no positive association with intentions to reduce food waste for their sample of Danish consumers.

The final retained component also features three significant loadings. The largest loading is positive (0.71) and denotes agreement with the statement that the respondent’s household wastes more food than similarly sized households. The second largest loading is negative (-0.66) and signals that respondents disagree that it is difficult to further reduce food waste (or, reversing the double negative, they may find it easy to further reduce food waste). The final significant loading is positive (0.45) and denotes agreement with the statement that buying in bulk or during sales can lead to greater food waste. This ‘Food Waste Reduction Potential’ component may represent a blend of constructs from the theories mentioned above. The component loads positively on agreement with the statement that the respondent’s household wastes more food than similarly sized households, yielding the clearest assessment of deviation from benchmark behavior. This suggests a possible link to a social norm, though the instrument doesn’t assess whether the respondent views deviation in the amount of food waste as a breach of social norms *per se*. The component loads negatively on agreement with a statement that it would be difficult for the household to further reduce food waste, which speaks to the construct of Perceived Behavioral Control (PBC) mentioned previously. Finally, a significant positive loading for the component comes from agreement that buying in bulk leads to more wasted food in their household. This may be a precursor to PBC as those who agree with this statement essentially admit to a consumption pattern that exacerbates food waste and, hence, acknowledge one possible means by which they can increase the pro-social behavior of reducing food waste.

PBC is influenced by past experience as well as perceived barriers of the behavior, which influences the perceived difficulty of engaging in the behavior. These perceptions of control may be influenced by benchmarking exercises in which households compare their behavior with that of other households. Such benchmarking has been shown to be relevant in pro-environmental behaviors such as energy conservation [28, 29] and may feature some conceptual spillover with social norm constructs. We note that Stancu, Haugaard and Lähteenmäki [27] features a PBC construct in their model, but they do not consider household benchmarking.

For each respondent, we predict each retained component score and then regress the predicted scores against the array of personal and household characteristics outlined earlier. Results are presented in Table 3 and feature several significant associations that merit discussion. Income is jointly significant in the regression for the first component and reveals that the highest income group is associated with the highest predicted scores for the ‘Practical Benefits’ component. For the ‘Guilt’ component the race category is jointly significant. Those identifying as Asian or identifying with other racial groups score highest on this component. Employment status and region of residence are jointly significant for the ‘Food Waste Reduction Potential’ component. Respondents who reside in the West scored lowest on this component as did those identifying as a student or homemaker.

**Table 3:** Regression models of principal components.

**Variables**		**Practical Benefits of Food Waste**		**Food Waste Guilt**		**Food Waste Reduction Potential**	
		Coef.	*p value*	Coef.	*p value*	Coef.	*p value*.
**Income**	<$50,000(Omitted)	Joint p = 0.021*^a^		*Joint p = 0*.*438*		*Joint p = 0*.*253*	
	$50,000-$99,999	-0.254	0.059	0.068	0.614	-0.127	0.368
	More than $100,000	0.178	0.281	-0.225	0.197	-0.134	0.425
	Refused/Missing	-0.310	0.133	0.030	0.874	-0.375*	0.047
**Race**	White, non-Hispanic (Omitted)	*Joint p = 0*.*233*		Joint p = 0.03*		*Joint p = 0*.*695*	
	Black, non-Hispanic	-0.185	0.268	0.088	0.630	0.0004	0.998
	Hispanic	0.324	0.153	0.001	0.997	0.158	0.501
	Other race	0.150	0.52	0.529*	0.005	0.347	0.283
**Employment**	Full-Time(Omitted)	*Joint p = 0*.*436*		*Joint p = 0*.*320*		Joint p = 0.011*	
	Part-time	-0.098	0.592	-0.135	0.419	0.306	0.083
	Retired	0.144	0.48	-0.021	0.915	0.018	0.937
	Homemaker	0.551	0.056	0.002	0.995	-0.434*	0.044
	Student	-0.213	0.518	-0.171	0.524	-0.722*	0.026
	Temporarily unemployed	-0.074	0.823	0.301	0.307	-0.226	0.444
	Disabled/Handicapped/Other not employed	-0.146	0.616	0.443*	0.039	-0.005	0.984
**Region**	North East (Omitted)	*Joint p = 0*.*793*		*Joint p = 0*.*145*		Joint p = 0.016*	
	North Central	0.170	0.370	0.003	0.986	0.094	0.622
	South	0.053	0.75	0.122	0.514	-0.117	0.539
	West	0.039	0.839	0.352	0.067	-0.394*	0.050
**FW Aware?**		-0.036	0.781	0.217	0.085	-0.211	0.074
**Metro Area**	Center City (Omitted)	*Joint p = 0*.*995*		*Joint p = 0*.*178*		*Joint p = 0*.*805*	
	Center City County	0.040	0.806	0.335*	0.026	0.121	0.470
	Suburban	-0.037	0.816	0.013	0.941	0.125	0.402
	Non-Center City	0.087	0.851	0.318	0.350	0.328	0.461
	Non-Metro	-0.007	0.961	0.008	0.964	-0.026	0.872
**Male**		0.020	0.88	-0.223	0.066	-0.144	0.245
**Homeowner**		-0.098	0.448	-0.045	0.730	-0.227	0.073
**Marital Status**	Single, that is never married (Omitted)	*Joint p = 0*.*072*		*Joint p = 0*.*456*		*Joint p = 0*.*098*	
	Single, live with a partner	-0.401	0.097	-0.262	0.323	-0.403	0.060
	Married	-0.535*	0.006	0.012	0.953	-0.333	0.102
	Separated	0.133	0.758	0.339	0.294	-0.289	0.476
	Widowed	-0.297	0.334	0.213	0.445	0.146	0.603
	Divorced	-0.260	0.247	-0.161	0.493	-0.413*	0.039
**Age**	18–29 (Omitted)	*Joint p = 0*.*098*		*Joint p = 0*.*165*		*Joint p = 0*.*423*	
	30–49	-0.020	0.922	-0.286	0.140	0.102	0.606
	50–64	0.342	0.108	-0.271	0.275	0.290	0.187
	65+	0.131	0.603	-0.579*	0.043	0.041	0.875
**Education**	<High School (Omitted)	*Joint p = 0*.*987*		*Joint p = 0*.*808*		*Joint p = 0*.*858*	
	High School	-0.060	0.788	0.300	0.303	0.159	0.513
	Some College	-0.087	0.717	0.245	0.434	0.244	0.315
	Graduate College	-0.035	0.88	0.202	0.534	0.184	0.492
	Graduate School	-0.115	0.636	0.347	0.323	0.250	0.339
**Religion**	1 (Omitted)	*Joint p = 0*.*297*		*Joint p = 0*.*669*		*Joint p = 0*.*285*	
	2	-0.113	0.467	0.210	0.191	-0.166	0.296
	3	-0.248	0.312	0.305	0.395	0.066	0.868
	4	-0.363	0.123	-0.048	0.844	-0.202	0.321
	5	-0.298	0.428	-0.113	0.780	-0.451	0.264
	6	-0.350*	0.039	0.109	0.534	0.149	0.388
**# Kids in HH**		0.119	0.28	0.083	0.413	0.034	0.758
**HH size**		0.071	0.468	-0.036	0.678	0.090	0.316
**# Adult Females in HH**		-0.134	0.249	0.081	0.404	-0.004	0.967
**R**^**2**^		0.153		0.163		0.182	

* *p*<5%.

Principal component scores are normalized and feature mean zero and a standard deviation of one. Hence the regression coefficients can be interpreted as effect sizes. For example, for the Practical Benefits component, the effect of being in the middle income group versus the lowest income group is associated with 0.254 standard deviation decline in the principal component while having one less child in a household is associated with a 0.119 standard deviation decline in the component.

^a^–denotes the p-value from the F-test statistic that this category’s regression coefficients are jointly equal to zero.

See the footnote to Table 2 for details of religious affiliation variable.

## Discussion and Implications

The congruence of several findings between our results and those in the literature suggests an emerging set of core stylized facts about U.S. consumer awareness and perceptions. There is an emerging sense that awareness of food waste is high but not nearly universal; that most households feel guilty about food waste; and that these general feelings of guilt about food waste clearly exceed specific concerns about negative effects of food waste on the environment. There also appears to be consensus that a majority of households find that enhancing meal safety and freshness may sometimes require wasting food. We look forward to future work that can continue to track and refine these findings and insights.

To our knowledge, we are the first to apply principal component analysis to attitudinal responses of U.S. consumers concerning food waste to form composite constructs. The three retained components align with theoretical constructs invoked to explain pro-social and pro-environmental behaviors elsewhere in the literature. We look forward to future research to see if others identify similar commonalities in attitudinal responses and how these are related to intended, self-report and objectively measured food waste behaviors. If a consistent set of components can be identified, it can facilitate the development of tractable behavioral models of consumer approaches to household food waste issues. Such models could inform the development and targeting of informational or other policy interventions that focus on these constellations of issues.

For example, in Table A in S1 File we identify that those who express awareness of food waste are significantly more likely to agree that they feel guilty when throwing away food, and this statement loads positively on the Food Waste Guilt component. Guilt, defined as “…the negative affective experience aroused when one’s behavior falls short of social or moral norms…” [37, 38], plays a critical role in self-regulation and functions as a motivator to keep behaviors in line with perceived standards [39]. Neff, Truant and Spiker note that 60% of their sample agree that they try to reduce waste because of guilt. Together, this could suggest a possible pathway to reduce food waste that begins with awareness, which generates guilt by delineating a moral norm that can then lead to household food waste reduction efforts. This hypothesis is supported by the norm-activation model [16] that regards moral norm as direct determinants of pro-environmental behaviors. The formation of a moral norm, which delivers the standard about what are the right things to do, is an interaction of cognitive, emotional and social factors. Awareness and knowledge about food waste are likely to be crucial cognitive antecedents for the development of moral norms. However, moral norms may not drive intended or actual behavior with respect to food waste, as Stancu, Haugaard and Lähteenmäki [31] find no significant association between their moral norm construct and intended food waste behavior for Danish consumers. Whether such associations are unique to Danish consumers or also apply to U.S. consumers in the food waste context must be addressed in future work.

Our results also point to another informational avenue that can build on this norm-based strategy to reduce food waste. Both our study and that of Neff, Truant and Spiker reveal that the vast majority of respondents view their food waste as average or less than average, which suggests that few people identify current behavior as a deviation from social norms. Interventions that measure a household’s food waste level and place it in perspective of societal averages or a socially-endorsed goal (benchmarking) could result in stronger norm activation, more positive attitudes towards reducing food waste, and stronger intentions to reduce food waste, which could lead to improved behaviors (we thank an anonymous reviewer for this insight). Interventions by energy companies that measure household energy usage and report the household’s usage compared to that of neighbors have yielded reductions in energy usage across all households involved [40, 41], though similarly effective household-level measurement technology is currently lacking in the realm of food waste.

The regression analysis in Table 3 also reveals that higher income households tend to score higher than other income groups on the Practical Benefits component, which indicates strong agreement by the respondents with the ideas that food waste is needed to ensure fresh, high quality meals, to reduce foodborne illness, and to economize on time. These insights align with economic intuition, in that higher income households have a high opportunity cost for their time (both household time for planning, shopping and preparation and time gained from avoided illnesses) and generally demand goods perceived to be higher quality (e.g., fresher meals). Given these general demand patterns among higher income households, informational treatments aimed at such households that communicate simple ways to circumvent the perceived freshness versus food waste trade off may stimulate lower food waste among such households. Also, such households may be particularly responsive to initiatives that remove or clarify food label dates, which may mitigate food labelling confusion and alleviate perceived trade-offs between food waste and foodborne illness [42].

According to the regression results shown in Table 3, those identified as the ‘other’ race (not White, not Black, not Hispanic) are significantly more likely to score higher than other groups on the principle component dubbed food waste guilt, implying agreement with linkages between throwing away food and guilt, environmental degradation, and wasted household money. Similar patterns are also found in agreement that more food is wasted when food is bought in bulk (Table E in S1 File). For those who identify as ‘other race,’ about half list ancestral affiliation with Asia, with most arriving from or having ancestors who arrived from developing countries where food or other resources may be scarce and social norms against wasting food or other resources may be stringent. Therefore, people from those countries may be more self-regulating and be more aware of the source of food waste. For example, Japan, South Korea, and Singapore, which are among the few developed economies in Asia, have very strict waste recycling systems because of the scarcity of natural resources. Indeed, the South Korean government charges residents per unit of food waste created [19]. Other developing countries, like China, may have strong cultural norms about saving food because of historical food shortages, and recent press coverage urges an end to food waste at business meals [43].

### Limitations

One limitation of this study is that we do not assess self-reported individual food waste behaviors, which often yield downwardly biased estimates of household food waste as consumers may tend to report what is closer to perceived moral norms than to the actual amount [44, 45]. Another limitation is our use of home ownership as a proxy for a household’s ability to invest in food storage. The response rate to this omnibus phone survey is 8%, and represents another of the study’s limitations. Phone surveys can yield higher response rates, but we relied upon the omnibus survey platform provided by our vender, where all recruited respondents must finish the survey during the same week they are first contacted. Due to limited funding, we also rely upon a smaller sample size than previous national surveys (500 versus 1000). Finally, both our work and that of Neff, Truant and Spiker rely on cross sectional (snapshot) data. Additional research that can track a panel of individuals over time or provide another source to identify causal linkages would be required to validate the pathways conjectured in this and other work.

We also note our ability to compare our sample’s awareness and attitudinal responses to those of Neff, Truant and Spiker’s sample faces some limitations. For example, it would be ideal to track any changes to U.S. consumer awareness of food waste issues in the 15 months between the two surveys. We find general awareness of food waste to be 11 percentage points higher in our sample than in Neff, Spiker and Truant’s sample, but differences in awareness could be related to several factors beyond population change. We employ a different sampling method (random digit dialing versus maintained consumer panel), administer the survey via a different interview mode (telephone versus online), and use different wording to assess key awareness and attitude constructs. For example, our awareness question was more broadly worded and mentions ‘…ways to reduce the amount of food wasted…’ while Neff, Spiker and Truant’s question is more narrowly focused on waste alone.

## Conclusions

If the United States is to reach its recently announced goal of reducing food waste by 50% by the year 2030, U.S. consumers must be an integral part of any successful plan, either by directly altering their household food waste behaviors or by inducing other actors in the food supply chain to reduce food waste. Our survey results suggest that the first step to engaging U.S. consumers–generating awareness of food waste–has surpassed the 50% mark. It also suggests that increasing public concern about the environmental threat posed by wasted food may be an important early step, as we document that specific concerns about the environmental harm posed by food waste lag considerably behind general feelings of non-specific guilt about food waste.

Food label guides or initiatives like the removal of sell-by dates could help reduce food label confusion and alleviate the perceived tradeoff between food waste and foodborne illness, which may be able to reduce some food waste efficiently. Such an information initiative could be especially effective among high income households and females who waste food because of health concerns but strongly feel guilty about food waste at the same time. While assessing consumer awareness, attitudes and opinions concerning food waste provides important information for assessing potential information and other policy interventions, additional work to refine and simplify physical measures of food waste at both the household and more aggregate levels will be critical for further in-depth consumer studies and improving the implementation and tracking of food waste reduction interventions.

## Supporting Information

S1 File **Table A:** Regression results: Guilty about food waste. **Table B:** Regression results: Food waste reduces foodborne illness. **Table C:** Regression results: Food waste ensures quality. **Table D:** Regression results: Food waste is bad for environment. **Table E:** Regression results: More food is wasted when buy in bulk. **Table F: Regression results:** Hard to reduce food waste further. **Table G:** Regression results: Food waste is a waste of money. **Table H:** Regression results: No time to worry about food waste. **Table I:** Regression results: Waste more food than others. **Table J:** Regression results: Responsibility for food shopping and meal preparation. **Table K:** Chi-Square test: food waste awareness and household characteristics. **Table L:** Chi-square test: Guilty about food waste. **Table M:** Chi-square test: Food waste reduces foodborne illness. **Table N:** Chi-square test: Food waste ensures quality. **Table O:** Chi-square test: Food waste is bad for environment. **Table P:** Chi-square test: More food is wasted when buy in bulk. **Table Q:** Chi-square test: Hard to reduce food waste further. **Table R:** Chi-square test: Food waste is a waste of money. **Table S:** Chi-square test: No time to worry about food waste. **Table T:** Chi-square test: Waste more food than others.
(DOCX)

S2 File Survey questionnaire. (DOC)

S3 File Survey data. (XLSX)
