# Supplementary material for: Household Food Waste: Multivariate Regression and Principal Components Analyses of Awareness and Attitudes among U.S. Consumers
Source: PLoS One. 2016 Jul 21;11(7):e0159250. doi: 10.1371/journal.pone.0159250 (PMC4956302; doi:10.1371/journal.pone.0159250)
Supplement: S1 File — Table A: Regression results: Guilty about food waste. Table B: Regression results: Food waste reduces foodborne illness. Table C: Regression results: Food waste ensures quality. Table D: Regression results: Food waste is bad for environment. Table E: Regression results: More food is wasted when buy in bulk. Table F: Regression results: Hard to reduce food waste further. Table G: Regression results: Food waste is a waste of money. Table H: Regression results: No time to worry about food waste. Table I: Regression results: Waste more food than others. Table J: Regression results: Responsibility for food shopping and meal preparation. Table K: Chi-Square test: food waste awareness and household characteristics. Table L: Chi-square test: Guilty about food waste. Table M: Chi-square test: Food waste reduces foodborne illness. Table N: Chi-square test: Food waste ensures quality. Table O: Chi-square test: Food waste is bad for environment. Table P: Chi-square test: More food is wasted when buy in bulk. Table Q: Chi-square test: Hard to reduce food waste further. Table R: Chi-square test: Food waste is a waste of money. Table S: Chi-square test: No time to worry about food waste. Table T: Chi-square test: Waste more food than others. (DOCX) [file pone.0159250.s001.docx]

**Table A: Regression results: Guilty about food waste**

| ***Feel guilty about FW^a^*** | | | |
| --- | --- | --- | --- |
|  |  | Coef. | *p*-value |
| **Income** |  | *Joint p=* *0.253* | |
|  | $50,000-$99,999 | 0.043 | 0.878 |
|  | More than $100,000 | -0.569 | 0.109 |
|  | Refused/Missing | 0.244 | 0.539 |
| **Education** |  | *Joint p=0.979* | |
|  | High School | -0.178 | 0.746 |
|  | Some College | -0.111 | 0.852 |
|  | Graduate College | -0.134 | 0.832 |
|  | Graduate School | 0.063 | 0.929 |
| **Age** |  | *Joint p=0.055* | |
|  | 30-49 | -0.484 | 0.224 |
|  | 50-64 | -0.280 | 0.502 |
|  | 65+ | -1.100* | 0.020 |
| **Metro Area** |  | *Joint p=0.040** | |
|  | Center City County | 0.799* | 0.030 |
|  | Surburban | -0.365 | 0.228 |
|  | Non-Center City | 0.961 | 0.231 |
|  | Non-Metro | -0.088 | 0.794 |
| **Homeowner** |  | 0.115 | 0.647 |
| **HH Size** |  | 0.045 | 0.833 |
| **Male** |  | -1.108* | 0.000 |
| **# Adult Females in HH** |  | -0.032 | 0.891 |
| **Race** |  | *Joint p=0.022** | |
|  | Black, non-Hispanic | 0.270 | 0.490 |
|  | Hispanic | -0.016 | 0.973 |
|  | Other race | 1.449* | 0.002 |
| **Employment** |  | *Joint p=0.561* | |
|  | Part-time | 0.002 | 0.997 |
|  | Retired | -0.386 | 0.295 |
|  | Homemaker | -0.798 | 0.063 |
|  | Student | -0.603 | 0.454 |
|  | Temporarily unemployed | 0.037 | 0.958 |
|  | Disabled/Handicapped/Other not employed | -0.216 | 0.683 |
| **Region** |  | *Joint p=0.648* | |
|  | North Central | 0.282 | 0.450 |
|  | South | 0.219 | 0.555 |
|  | West | 0.531 | 0.208 |
| **Marital Status** |  | *Joint p=0.871* | |
|  | Single, live with a partner | 0.169 | 0.806 |
|  | Married | -0.110 | 0.786 |
|  | Separated | 0.011 | 0.987 |
|  | Widowed | 0.077 | 0.887 |
|  | Divorced | -0.457 | 0.353 |
| **Religion** |  | *Joint p=0.773* | |
|  | 2 | 0.334 | 0.261 |
|  | 3 | 0.589 | 0.485 |
|  | 4 | 0.104 | 0.858 |
|  | 5 | 0.140 | 0.883 |
|  | 6 | -0.055 | 0.867 |
| **# Kids in HH** |  | -0.067 | 0.769 |
| **FW^a^ Aware?** |  | 0.506* | 0.037 |

***** denotes that regression coefficient is significantly different from zero at the 5% level.

Omitted categories for categorical variable include: Income – Less than $50,000; Education– less than high school; Age – 18 – 34; Metro Area– center city; Race– white not-Hispanic; Employment– Full-time job; Region– North East; Marital Status– Single, that is never married; Religion– Group1; Political ID– Republican. Religious categories are as follows: 1 (omitted) includes Baptist, Christian, Church of Christ, Church of God, Evangelical, Holiness, Non-denominational or Independent, Pentecostal; 2 includes Catholic, Congregational or UCC, Episcopalian or Anglican, Lutheran, Methodist, Orthodox, Presbyterian, Unitarian/Universalist, Protestant, 3 includes Buddhist, Hindu, Muslim/Islam; 4 includes Jehovah’s witness, Mormon, Seventh-Day Adventist; 5 includes Jewish; 6 includes Atheist, Agnostic, Other, Nothing in particular, Don’t know, Didn’t report.

**Table B: Regression results: Food waste reduces foodborne illness**

| ***FW reduces FB*^b^ *illness*** | | | |
| --- | --- | --- | --- |
|  |  | Coef. | *p*-value |
| **Income** |  | *Joint p=0.558* | |
|  | $50,000-$99,999 | -0.118 | 0.684 |
|  | More than $100,000 | 0.337 | 0.335 |
|  | Refused/Missing | -0.277 | 0.523 |
| **Education** |  | *Joint p=0.277* | |
|  | High School | 0.230 | 0.627 |
|  | Some College | -0.245 | 0.625 |
|  | Graduate College | -0.084 | 0.870 |
|  | Graduate School | -0.504 | 0.333 |
| **Age** |  | *Joint p=0.292* | |
|  | 30-49 | -0.192 | 0.612 |
|  | 50-64 | -0.218 | 0.592 |
|  | 65+ | -0.871 | 0.092 |
| **Metro Area** |  | *Joint p=0.358* | |
|  | Center City County | -0.429 | 0.188 |
|  | Surburban | -0.133 | 0.677 |
|  | Non-Center City | -0.776 | 0.341 |
|  | Non-Metro | -0.508 | 0.090 |
| **Homeowner** |  | -0.234 | 0.365 |
| **HH Size** |  | 0.166 | 0.345 |
| **Male** |  | 0.166 | 0.488 |
| **# Adult Females in HH** |  | -0.197 | 0.381 |
| **Race** |  | *Joint p=0.178* | |
|  | Black, non-Hispanic | 0.012 | 0.974 |
|  | Hispanic | 0.918 | 0.084 |
|  | Other race | 0.723 | 0.099 |
| **Employment** |  | *Joint p=0.349* | |
|  | Part-time | -0.385 | 0.212 |
|  | Retired | -0.113 | 0.774 |
|  | Homemaker | 0.681 | 0.247 |
|  | Student | 0.001 | 0.999 |
|  | Temporarily unemployed | -0.878 | 0.075 |
|  | Disabled/Handicapped/Other not employed | 0.319 | 0.653 |
| **Region** |  | *Joint p=0.726* | |
|  | North Central | 0.224 | 0.510 |
|  | South | 0.275 | 0.415 |
|  | West | 0.389 | 0.258 |
| **Marital Status** |  | *Joint p=0.648* | |
|  | Single, live with a partner | -0.368 | 0.416 |
|  | Married | -0.231 | 0.498 |
|  | Separated | 0.392 | 0.536 |
|  | Widowed | 0.428 | 0.479 |
|  | Divorced | -0.030 | 0.947 |
| **Religion** |  | *Joint p=*0.378 | |
|  | 2 | 0.270 | 0.399 |
|  | 3 | -1.052 | 0.145 |
|  | 4 | 0.165 | 0.738 |
|  | 5 | 0.601 | 0.245 |
|  | 6 | 0.055 | 0.870 |
| **# Kids in HH** |  | 0.001 | 0.994 |
| **FW^a^ Aware?** |  | 0.025 | 0.919 |

***** denotes that regression coefficient is significantly different from zero at the 5% level.

**Table C: Regression results: Food waste ensures quality**

| ***FW^a^ ensures quality*** | | | |
| --- | --- | --- | --- |
|  |  | Coef. | *p*-value |
| **Income** |  | *Joint p=0.087* | |
|  | $50,000-$99,999 | -0.585* | 0.030 |
|  | More than $100,000 | 0.096 | 0.754 |
|  | Refused/Missing | -0.400 | 0.294 |
| **Education** |  | *Joint p=0.585* | |
|  | High School | -0.619 | 0.166 |
|  | Some College | -0.394 | 0.408 |
|  | Graduate College | -0.420 | 0.388 |
|  | Graduate School | -0.212 | 0.671 |
| **Age** |  | *Joint p=0.028** | |
|  | 30-49 | 0.098 | 0.817 |
|  | 50-64 | 0.761 | 0.095 |
|  | 65+ | -0.015 | 0.978 |
| **Metro Area** |  | *Joint p=0.675* | |
|  | Center City County | -0.024 | 0.940 |
|  | Surburban | -0.428 | 0.171 |
|  | Non-Center City | 0.275 | 0.752 |
|  | Non-Metro | -0.146 | 0.617 |
| **Homeowner** |  | -0.098 | 0.704 |
| **HH Size** |  | 0.155 | 0.427 |
| **Male** |  | -0.460 | 0.053 |
| **# Adult Females in HH** |  | -0.481 | 0.057 |
| **Race** |  | *Joint p=0.196* | |
|  | Black, non-Hispanic | -0.251 | 0.474 |
|  | Hispanic | 0.613 | 0.122 |
|  | Other race | 0.610 | 0.176 |
| **Employment** |  | *Joint p=0.371* | |
|  | Part-time | -0.412 | 0.215 |
|  | Retired | 0.537 | 0.157 |
|  | Homemaker | 0.493 | 0.389 |
|  | Student | -0.531 | 0.397 |
|  | Temporarily unemployed | 0.231 | 0.711 |
|  | Disabled/Handicapped/Other not employed/ | -0.200 | 0.684 |
| **Region** |  | *Joint p=0.083* | |
|  | North Central | 0.126 | 0.716 |
|  | South | -0.128 | 0.695 |
|  | West | -0.683 | 0.059 |
| **Marital Status** |  | *Joint p=0.398* | |
|  | Single, live with a partner | -0.360 | 0.518 |
|  | Married | -0.580 | 0.152 |
|  | Separated | 0.615 | 0.488 |
|  | Widowed | -0.167 | 0.772 |
|  | Divorced | -0.681 | 0.106 |
| **Religion** |  | *Joint p=0.324* | |
|  | 2 | -0.180 | 0.533 |
|  | 3 | -0.395 | 0.595 |
|  | 4 | -0.842 | 0.125 |
|  | 5 | 0.627 | 0.381 |
|  | 6 | -0.459 | 0.144 |
| **# Kids in HH** |  | -0.032 | 0.880 |
| **FW^a^ Aware?** |  | 0.049 | 0.844 |

***** denotes that regression coefficient is significantly different from zero at the 5% level.

**Table D: Regression results: Food waste is bad for environment**

| ***FW^a^ bad for environment*** | | | |
| --- | --- | --- | --- |
|  |  | Coef. | *p*-value |
| **Income** |  | *Joint p=0.345* | |
|  | $50,000-$99,999 | -0.006 | 0.981 |
|  | More than $100,000 | -0.550 | 0.101 |
|  | Refused/Missing | -0.062 | 0.896 |
| **Education** |  | *Joint p=0.041** | |
|  | High School | 0.490 | 0.354 |
|  | Some College | 0.608 | 0.266 |
|  | Graduate College | 0.737 | 0.205 |
|  | Graduate School | 1.591* | 0.011 |
| **Age** |  | *Joint p=0.149* | |
|  | 30-49 | -0.842* | 0.040 |
|  | 50-64 | -0.657 | 0.171 |
|  | 65+ | -1.079 | 0.056 |
| **Metro Area** |  | *Joint p=0.887* | |
|  | Center City County | 0.230 | 0.445 |
|  | Surburban | 0.139 | 0.674 |
|  | Non-Center City | 0.282 | 0.696 |
|  | Non-Metro | -0.077 | 0.800 |
| **Homeowner** |  | -0.036 | 0.889 |
| **HH Size** |  | -0.029 | 0.844 |
| **Male** |  | -0.141 | 0.531 |
| **# Adult Females in HH** |  | 0.134 | 0.525 |
| **Race** |  | *Joint p=0.491* | |
|  | Black, non-Hispanic | -0.151 | 0.668 |
|  | Hispanic | 0.390 | 0.397 |
|  | Other race | 0.516 | 0.233 |
| **Employment** |  | *Joint p=0.138* | |
|  | Part-time | -0.287 | 0.327 |
|  | Retired | 0.116 | 0.790 |
|  | Homemaker | 0.542 | 0.388 |
|  | Student | -0.151 | 0.784 |
|  | Temporarily unemployed | 0.604 | 0.327 |
|  | Disabled/Handicapped/Other not employed | 1.790* | 0.009 |
| **Region** |  | *Joint p=0.154* | |
|  | North Central | -0.355 | 0.334 |
|  | South | 0.096 | 0.772 |
|  | West | 0.337 | 0.359 |
| **Marital Status** |  | *Joint p=0.451* | |
|  | Single, live with a partner | -0.849 | 0.099 |
|  | Married | 0.132 | 0.749 |
|  | Separated | 0.404 | 0.531 |
|  | Widowed | 0.032 | 0.959 |
|  | Divorced | -0.080 | 0.854 |
| **Religion** |  | *Joint p=0.845* | |
|  | 2 | 0.139 | 0.593 |
|  | 3 | -0.003 | 0.996 |
|  | 4 | -0.473 | 0.422 |
|  | 5 | -0.642 | 0.661 |
|  | 6 | 0.222 | 0.481 |
| **# Kids in HH** |  | -0.006 | 0.974 |
| **FW^a^ Aware?** |  | 0.379 | 0.108 |

***** denotes that regression coefficient is significantly different from zero at the 5% level.

**Table E: Regression results: More food is wasted when buy in bulk**

| ***More FW^a^ when buy in bulk*** | | | |
| --- | --- | --- | --- |
|  |  | Coef. | *p*-value |
| **Income** |  | *Joint p=0.369* | |
|  | $50,000-$99,999 | -0.135 | 0.591 |
|  | More than $100,000 | 0.102 | 0.741 |
|  | Refused/Missing | -0.640 | 0.117 |
| **Education** |  | *Joint p=0.134* | |
|  | High School | 1.145* | 0.011 |
|  | Some College | 0.998* | 0.023 |
|  | Graduate College | 0.821 | 0.082 |
|  | Graduate School | 1.063* | 0.033 |
| **Age** |  | *Joint p=0.872* | |
|  | 30-49 | -0.220 | 0.530 |
|  | 50-64 | -0.087 | 0.834 |
|  | 65+ | -0.338 | 0.523 |
| **Metro Area** |  | *Joint p=0.825* | |
|  | Center City County | 0.144 | 0.642 |
|  | Surburban | -0.194 | 0.486 |
|  | Non-Center City | 0.218 | 0.762 |
|  | Non-Metro | 0.116 | 0.704 |
| **Homeowner** |  | -0.462 | 0.063 |
| **HH Size** |  | 0.099 | 0.638 |
| **Male** |  | -0.196 | 0.419 |
| **# Adult Females in HH** |  | -0.076 | 0.745 |
| **Race** |  | *Joint p=0.008** | |
|  | Black, non-Hispanic | -0.366 | 0.334 |
|  | Hispanic | 0.256 | 0.440 |
|  | Other race | 1.468* | 0.002 |
| **Employment** |  | *Joint p=0.363* | |
|  | Part-time | 0.061 | 0.860 |
|  | Retired | 0.123 | 0.781 |
|  | Homemaker | -0.930 | 0.099 |
|  | Student | -0.973 | 0.124 |
|  | Temporarily unemployed | -0.002 | 0.998 |
|  | Disabled/Handicapped/Other not employed | -0.462 | 0.507 |
| **Region** |  | *Joint p=0.877* | |
|  | North Central | 0.197 | 0.566 |
|  | South | 0.239 | 0.431 |
|  | West | 0.111 | 0.748 |
| **Marital Status** |  | *Joint p=0.240* | |
|  | Single, live with a partner | -0.790 | 0.118 |
|  | Married | -0.300 | 0.375 |
|  | Separated | 0.775 | 0.349 |
|  | Widowed | 0.424 | 0.396 |
|  | Divorced | -0.456 | 0.305 |
| **Religion** |  | *Joint p=0.975* | |
|  | 2 | -0.148 | 0.612 |
|  | 3 | -0.208 | 0.751 |
|  | 4 | -0.296 | 0.749 |
|  | 5 | -0.531 | 0.418 |
|  | 6 | -0.064 | 0.831 |
| **# Kids in HH** |  | 0.085 | 0.742 |
| **FW^a^ Aware?** |  | -0.084 | 0.713 |

***** denotes that regression coefficient is significantly different from zero at the 5% level.

**Table F: Regression results: Hard to reduce food waste further**

| ***Hard to reduce FW^a^ further*** | | | |
| --- | --- | --- | --- |
|  |  | Coef. | *p*-value |
| **Income** |  | *Joint p=0.157* | |
|  | $50,000-$99,999 | -0.377 | 0.201 |
|  | More than $100,000 | 0.164 | 0.641 |
|  | Refused/Missing | 0.366 | 0.286 |
| **Education** |  | *Joint p=0.982* | |
|  | High School | 0.097 | 0.829 |
|  | Some College | -0.087 | 0.849 |
|  | Graduate College | 0.033 | 0.941 |
|  | Graduate School | -0.087 | 0.869 |
| **Age** |  | *Joint p=0.692* | |
|  | 30-49 | 0.007 | 0.989 |
|  | 50-64 | -0.123 | 0.772 |
|  | 65+ | 0.363 | 0.493 |
| **Metro Area** |  | *Joint p=0.271* | |
|  | Center City County | 0.111 | 0.730 |
|  | Surburban | -0.279 | 0.358 |
|  | Non-Center City | -0.765 | 0.278 |
|  | Non-Metro | 0.388 | 0.230 |
| **Homeowner** |  | 0.219 | 0.390 |
| **HH Size** |  | -0.185 | 0.311 |
| **Male** |  | 0.139 | 0.589 |
| **# Adult Females in HH** |  | 0.234 | 0.302 |
| **Race** |  | *Joint p=0.632* | |
|  | Black, non-Hispanic | -0.443 | 0.225 |
|  | Hispanic | 0.107 | 0.806 |
|  | Other race | -0.103 | 0.851 |
| **Employment** |  | *Joint p=0.458* | |
|  | Part-time | -0.359 | 0.341 |
|  | Retired | 0.297 | 0.504 |
|  | Homemaker | 0.601 | 0.255 |
|  | Student | 0.574 | 0.364 |
|  | Temporarily unemployed | 0.233 | 0.660 |
|  | Disabled/Handicapped/Other not employed | -0.267 | 0.626 |
| **Region** |  | *Joint p=0.235* | |
|  | North Central | 0.068 | 0.847 |
|  | South | 0.003 | 0.994 |
|  | West | 0.578 | 0.152 |
| **Marital Status** |  | *Joint p=0.427* | |
|  | Single, live with a partner | 0.053 | 0.910 |
|  | Married | -0.413 | 0.340 |
|  | Separated | -0.108 | 0.874 |
|  | Widowed | -1.009 | 0.083 |
|  | Divorced | -0.070 | 0.883 |
| **Religion** |  | *Joint p=0.128* | |
|  | 2 | -0.277 | 0.380 |
|  | 3 | -0.510 | 0.616 |
|  | 4 | -0.447 | 0.211 |
|  | 5 | -0.749 | 0.111 |
|  | 6 | -0.938* | 0.007 |
| **# Kids in HH** |  | 0.242 | 0.201 |
| **FW^a^ Aware?** |  | 0.412 | 0.073 |

***** denotes that regression coefficient is significantly different from zero at the 5% level.

**Table G: Regression results: Food waste is a waste of money**

| ***FW*^a^ *is a waste of money*** | | | |
| --- | --- | --- | --- |
|  |  | Coef. | *p*-value |
| **Income** |  | *Joint p=0.457* | |
|  | $50,000-$99,999 | -0.285 | 0.339 |
|  | More than $100,000 | -0.391 | 0.270 |
|  | Refused/Missing | -0.558 | 0.191 |
| **Education** |  | *Joint p=0.341* | |
|  | High School | 0.859 | 0.109 |
|  | Some College | 0.804 | 0.132 |
|  | Graduate College | 0.868 | 0.100 |
|  | Graduate School | 0.377 | 0.521 |
| **Age** |  | *Joint p=0.104* | |
|  | 30-49 | 0.023 | 0.957 |
|  | 50-64 | 0.390 | 0.355 |
|  | 65+ | -0.407 | 0.420 |
| **Metro Area** |  | *Joint p=0.544* | |
|  | Center City County | 0.569 | 0.103 |
|  | Surburban | 0.091 | 0.773 |
|  | Non-Center City | 0.463 | 0.459 |
|  | Non-Metro | 0.217 | 0.515 |
| **Homeowner** |  | -0.473 | 0.088 |
| **HH Size** |  | 0.219 | 0.209 |
| **Male** |  | 0.030 | 0.901 |
| **# Adult Females in HH** |  | -0.177 | 0.413 |
| **Race** |  | *Joint p=0.213* | |
|  | Black, non-Hispanic | 0.409 | 0.260 |
|  | Hispanic | -0.039 | 0.932 |
|  | Other race | 0.873 | 0.067 |
| **Employment** |  | *Joint p=0.248* | |
|  | Part-time | -0.115 | 0.774 |
|  | Retired | 0.204 | 0.597 |
|  | Homemaker | 0.538 | 0.422 |
|  | Student | -0.763 | 0.180 |
|  | Temporarily unemployed | 0.924 | 0.082 |
|  | Disabled/Handicapped/Other not employed | 0.904 | 0.122 |
| **Region** |  | *Joint p=0.802* | |
|  | North Central | 0.283 | 0.368 |
|  | South | 0.097 | 0.776 |
|  | West | 0.061 | 0.864 |
| **Marital Status** |  | *Joint p=0.003** | |
|  | Single, live with a partner | -0.522 | 0.325 |
|  | Married | -0.395 | 0.323 |
|  | Separated | 0.606 | 0.476 |
|  | Widowed | 0.797 | 0.127 |
|  | Divorced | -0.813 | 0.056 |
| **Religion** |  | *Joint p=0.121* | |
|  | 2 | -0.033 | 0.919 |
|  | 3 | 1.509* | 0.045 |
|  | 4 | -1.127 | 0.054 |
|  | 5 | -0.403 | 0.717 |
|  | 6 | -0.122 | 0.720 |
| **# Kids in HH** |  | 0.136 | 0.523 |
| **FW^a^ Aware?** |  | -0.186 | 0.458 |

***** denotes that regression coefficient is significantly different from zero at the 5% level.

**Table H: Regression results: No time to worry about food waste**

| ***No time to worry about FW*^a^** | | | |
| --- | --- | --- | --- |
|  |  | Coef. | *p*-value |
| **Income** |  | *Joint p=0.272* | |
|  | $50,000-$99,999 | -0.346 | 0.211 |
|  | More than $100,000 | 0.249 | 0.479 |
|  | Refused/Missing | -0.175 | 0.644 |
| **Education** |  | *Joint p=0.720* | |
|  | High School | -0.490 | 0.252 |
|  | Some College | -0.634 | 0.164 |
|  | Graduate College | -0.446 | 0.323 |
|  | Graduate School | -0.396 | 0.452 |
| **Age** |  | *Joint p=0.475* | |
|  | 30-49 | 0.123 | 0.745 |
|  | 50-64 | 0.534 | 0.211 |
|  | 65+ | 0.144 | 0.773 |
| **Metro Area** |  | *Joint p=0.960* | |
|  | Center City County | 0.096 | 0.763 |
|  | Surburban | -0.033 | 0.913 |
|  | Non-Center City | 0.140 | 0.824 |
|  | Non-Metro | -0.190 | 0.541 |
| **Homeowner** |  | -0.059 | 0.808 |
| **HH Size** |  | 0.312 | 0.140 |
| **Male** |  | 0.086 | 0.738 |
| **# Adult Females in HH** |  | -0.196 | 0.396 |
| **Race** |  | *Joint p=0.619* | |
|  | Black, non-Hispanic | -0.195 | 0.594 |
|  | Hispanic | -0.401 | 0.367 |
|  | Other race | -0.475 | 0.323 |
| **Employment** |  | *Joint p=0.006** | |
|  | Part-time | 0.651 | 0.078 |
|  | Retired | -0.259 | 0.528 |
|  | Homemaker | 0.904 | 0.055 |
|  | Student | -0.836 | 0.167 |
|  | Temporarily unemployed | -1.386 | 0.074 |
|  | Disabled/Handicapped/Other not employed | -1.247* | 0.049 |
| **Region** |  | *Joint p=0.922* | |
|  | North Central | 0.243 | 0.501 |
|  | South | 0.096 | 0.764 |
|  | West | 0.089 | 0.811 |
| **Marital Status** |  | *Joint p=0.035* | |
|  | Single, live with a partner | -1.183* | 0.019 |
|  | Married | -1.112* | 0.004 |
|  | Separated | -0.353 | 0.642 |
|  | Widowed | -0.250 | 0.644 |
|  | Divorced | -0.606 | 0.189 |
| **Religion** |  | *Joint p=0.415* | |
|  | 2 | -0.181 | 0.547 |
|  | 3 | 0.068 | 0.945 |
|  | 4 | -0.055 | 0.930 |
|  | 5 | -1.053 | 0.340 |
|  | 6 | -0.614* | 0.043 |
| **# Kids in HH** |  | -0.101 | 0.650 |
| **FW^a^ Aware?** |  | -0.457 | 0.068 |

***** denotes that regression coefficient is significantly different from zero at the 5% level.

**Table I: Regression results: Waste more food than others**

| ***More FW*^a^ *than others*** | | | |
| --- | --- | --- | --- |
|  |  | Coef. | *p*-value |
| **Income** |  | *Joint p=0.824* | |
|  | $50,000-$99,999 | -0.013 | 0.967 |
|  | More than $100,000 | 0.094 | 0.801 |
|  | Refused/Missing | -0.374 | 0.406 |
| **Education** |  | *Joint p=0.646* | |
|  | High School | -0.789 | 0.160 |
|  | Some College | -0.496 | 0.365 |
|  | Graduate College | -0.650 | 0.258 |
|  | Graduate School | -0.330 | 0.604 |
| **Age** |  | *Joint p=0.283* | |
|  | 30-49 | -0.502 | 0.248 |
|  | 50-64 | 0.063 | 0.899 |
|  | 65+ | -0.525 | 0.351 |
| **Metro Area** |  | *Joint p=0.546* | |
|  | Center City County | 0.266 | 0.485 |
|  | Surburban | 0.097 | 0.779 |
|  | Non-Center City | 1.259 | 0.124 |
|  | Non-Metro | -0.125 | 0.705 |
| **Homeowner** |  | 0.124 | 0.680 |
| **HH Size** |  | 0.153 | 0.472 |
| **Male** |  | -0.060 | 0.829 |
| **# Adult Females in HH** |  | 0.132 | 0.644 |
| **Race** |  | *Joint p=0.203* | |
|  | Black, non-Hispanic | -0.962* | 0.040 |
|  | Hispanic | -0.425 | 0.444 |
|  | Other race | -0.410 | 0.634 |
| **Employment** |  | *Joint p=0.757* | |
|  | Part-time | -0.096 | 0.807 |
|  | Retired | -0.213 | 0.638 |
|  | Homemaker | -0.592 | 0.230 |
|  | Student | -0.365 | 0.577 |
|  | Temporarily unemployed | -0.710 | 0.395 |
|  | Disabled/Handicapped/Other not employed | 0.329 | 0.519 |
| **Region** |  | *Joint p=0.003** | |
|  | North Central | -0.126 | 0.722 |
|  | South | -0.829* | 0.023 |
|  | West | -1.239* | 0.003 |
| **Marital Status** |  | *Joint p=0.167* | |
|  | Single, live with a partner | -1.310* | 0.025 |
|  | Married | -0.833 | 0.070 |
|  | Separated | -0.141 | 0.876 |
|  | Widowed | -0.347 | 0.616 |
|  | Divorced | -0.229 | 0.624 |
| **Religion** |  | *Joint p=0.060* | |
|  | 2 | -0.813* | 0.010 |
|  | 3 | -1.315 | 0.151 |
|  | 4 | -1.343* | 0.039 |
|  | 5 | -1.280 | 0.333 |
|  | 6 | -0.401 | 0.259 |
| **# Kids in HH** |  | 0.306 | 0.283 |
| **FW^a^ Aware?** |  | -0.267 | 0.327 |

***** denotes that regression coefficient is significantly different from zero at the 5% level.

**Table J: Regression results: Responsibility for food shopping and meal preparation**

| ***Responsibility for food shopping and meal preparation*** | | | |
| --- | --- | --- | --- |
|  |  | Coef. | *p*-value |
| **Income** |  | *Joint p=0.003** | |
|  | $50,000-$99,999 | 0.997* | 0.001 |
|  | More than $100,000 | 0.553 | 0.131 |
|  | Refused/Missing | -0.427 | 0.328 |
| **Education** |  | *Joint p=0.101* | |
|  | High School | 0.408 | 0.397 |
|  | Some College | 0.482 | 0.331 |
|  | Graduate College | -0.468 | 0.412 |
|  | Graduate School | -0.141 | 0.810 |
| **Age** |  | *Joint p=0.105* | |
|  | 30-49 | -0.200 | 0.629 |
|  | 50-64 | -0.793 | 0.079 |
|  | 65+ | 0.129 | 0.836 |
| **Metro Area** |  | *Joint p=0.102* | |
|  | Center City County | 0.263 | 0.465 |
|  | Suburban | -0.608* | 0.050 |
|  | Non-Center City | -0.859 | 0.269 |
|  | Non-Metro | -0.425 | 0.248 |
| **Homeowner** |  | 0.414 | 0.143 |
| **HH Size** |  | 0.209 | 0.233 |
| **Male** |  | 1.719* | 0.000 |
| **# Adult Females in HH** |  | 0.360 | 0.254 |
| **Race** |  | *Joint p=0.288* | |
|  | Black, non-Hispanic | -0.364 | 0.401 |
|  | Hispanic | -0.816 | 0.061 |
|  | Other race | -0.160 | 0.732 |
| **Employment** |  | *Joint p=0.025** | |
|  | Part-time | 0.187 | 0.629 |
|  | Retired | -0.112 | 0.832 |
|  | Homemaker | -0.812 | 0.199 |
|  | Student | 0.635 | 0.288 |
|  | Temporarily unemployed | 0.881 | 0.159 |
|  | Disabled/Handicapped/Other not employed | 1.819* | 0.002 |
| **Region** |  | *Joint p=0.512* | |
|  | North Central | 0.466 | 0.242 |
|  | South | 0.361 | 0.331 |
|  | West | 0.039 | 0.927 |
| **Marital Status** |  | *Joint p=0.205* | |
|  | Single, live with a partner | 0.055 | 0.924 |
|  | Married | 0.446 | 0.234 |
|  | Separated | -0.353 | 0.580 |
|  | Widowed | 0.312 | 0.612 |
|  | Divorced | -0.673 | 0.208 |
| **Religion** |  | *Joint p=0.139* | |
|  | 2 | -0.455 | 0.193 |
|  | 3 | 0.578 | 0.482 |
|  | 4 | -0.405 | 0.556 |
|  | 5 | 1.269 | 0.065 |
|  | 6 | -0.078 | 0.839 |
| **# Kids in HH** |  | -0.006 | 0.976 |

***** denotes that regression coefficient is significantly different from zero at the 5% level.

**Table K Chi-Square test: food waste awareness and household characteristics.**

| **FW^a^ Awareness** | | | | |
| --- | --- | --- | --- | --- |
| **Variable** | | **No**  **(%)** | **Uncertain**  **(%)** | **Yes**  **(%)** |
| **All** |  | 39.48 | 7.82 | 52.69 |
| **Education** | <High School | 52.11* | 14.37* | 33.52* |
|  | High School | 39.62* | 9.18* | 51.2* |
|  | Some College | 34.94* | 6.85* | 58.21* |
|  | Graduate College | 42.11* | 5.73* | 52.16* |
|  | Graduate School | 34.64* | 3.4* | 61.96* |
| **Income** | Refused/Missing | 46.07 | 3.92 | 50.01 |
|  | Less than $50,000 | 39.77 | 8.93 | 51.29 |
|  | $50,000-$99,999 | 37.18 | 8.52 | 54.29 |
|  | More than $100,000 | 38.26 | 5.11 | 56.64 |
| **Age** | 18-29 | 41.96* | 4.93* | 53.11* |
|  | 30-49 | 45.83* | 10.71* | 43.47* |
|  | 50-64 | 30.37* | 8.93* | 60.7* |
|  | 65+ | 38.08* | 4.46* | 57.45* |
| **Gender** | Male | 37.83 | 8.41 | 53.76 |
|  | Female | 41.04 | 7.27 | 51.69 |
| **Race** | White, non-Hispanic | 38.46* | 6.49* | 55.04* |
|  | Black, non-Hispanic | 44.30* | 14.40* | 41.30* |
|  | Hispanic | 41.67* | 13.41* | 44.93* |
|  | Other race | 38.10* | 0.00 * | 61.90* |
| **Kids** | No kids | 36.47* | 5.01* | 58.52* |
|  | Have kids | 44.37* | 12.38* | 43.24* |
| **Household Size** | 1-2 | 37.17* | 4.58* | 58.25* |
|  | 3-4 | 41.85* | 5.45* | 52.69* |
|  | 5+ | 37.17* | 23.52* | 39.30* |

Highlighted denotes p<0.05

**Table L: Chi-square test: Guilty about food waste**

| ***Feel guilty about FW*^a^** | | | | | |
| --- | --- | --- | --- | --- | --- |
|  |  | **Agree Strongly** | **Agree Somewhat** | **Diagree Somewhat** | **Diagree Strongly** |
| **All** |  | 55.81 | 21.37 | 9.47 | 13.34 |
| **Education** | <High School | 59.66 | 14.22 | 3.46 | 22.66 |
|  | High School | 52.42 | 24.87 | 11.28 | 11.43 |
|  | Some College | 57.65 | 19.94 | 8.36 | 14.05 |
|  | Graduate College | 47.32 | 28.18 | 14.48 | 10.02 |
|  | Graduate School | 65.66 | 15.13 | 7.28 | 11.94 |
| **Household Income** | Refused/Missing | 63.79 | 16.2 | 11.39 | 8.62 |
|  | Less than $50,000 | 56.02 | 19.39 | 9.14 | 15.45 |
|  | $50,000-$99,999 | 56.81 | 19.24 | 12.96 | 11 |
|  | More than $100,000 | 49.64 | 33.71 | 4.68 | 11.97 |
| **Age** | 18-29 | 61.77 | 20.01 | 9.94 | 8.28 |
|  | 30-49 | 55.13 | 24.87 | 9.07 | 10.92 |
|  | 50-64 | 60 | 19.77 | 6.7 | 13.53 |
|  | 65+ | 44.3 | 19.11 | 13.22 | 23.37 |
| **Gender** | Male | 47.66* | 22.22* | 12.68* | 17.45* |
|  | Female | 63.19* | 20.61* | 6.57* | 9.63* |
| **Race** | White, non-Hispanic | 52.25 | 22.34 | 11.14 | 14.27 |
|  | Black, non-Hispanic | 59.07 | 23.89 | 5.32 | 11.73 |
|  | Hispanic | 60.98 | 17.77 | 4.61 | 16.64 |
|  | Other race | 70.57 | 20.07 | 7.11 | 2.25 |
| **Kids** | No kids | 55.65 | 19.04 | 10.21 | 15.11 |
|  | Have kids | 56.08 | 25.2 | 8.27 | 10.46 |
| **Household Size** | 1--2 | 51.58 | 21.94 | 11.15 | 15.33 |
|  | 3--4 | 57.74 | 21.4 | 6.93 | 13.93 |
|  | 5+ | 65.5 | 19.12 | 8.19 | 7.19 |

Highlighted denotes p<0.05

**Table M: Chi-square test: Food waste reduces foodborne illness**

| ***FW*^a^ *reduces FB*^b^ *illness*** | | | | | |
| --- | --- | --- | --- | --- | --- |
|  |  | **Agree Strongly** | **Agree Somewhat** | **Diagree Somewhat** | **Diagree Strongly** |
| **All** |  | 40.97 | 28.73 | 19.78 | 10.51 |
| **Education** | <High School | 59.17* | 14.9* | 8.29* | 17.64* |
|  | High School | 46.19* | 25.24* | 21.03* | 7.55* |
|  | Some College | 35.05* | 34.33* | 19.77* | 10.85* |
|  | Graduate College | 39.64* | 28.67* | 20.23* | 11.45* |
|  | Graduate School | 25.3* | 38.39* | 27.25* | 9.06* |
| **Household Income** | Refused/Missing | 43.4* | 19.06* | 17.76* | 19.79* |
|  | Less than $50,000 | 44.24* | 23.7* | 20.03* | 12.03* |
|  | $50,000-$99,999 | 33.08* | 38.64* | 22.12* | 6.16* |
|  | More than $100,000 | 39.76* | 36.89* | 16.73* | 6.62* |
| **Age** | 18-29 | 44.5* | 34.26* | 13.97* | 7.27* |
|  | 30-49 | 45.93* | 28.08* | 20.44* | 5.55* |
|  | 50-64 | 39.16* | 30.24* | 18.54* | 12.06* |
|  | 65+ | 30.96* | 21.04* | 26.95* | 21.05* |
| **Gender** | Male | 44.24 | 27.58 | 18.1 | 10.08 |
|  | Female | 37.83 | 29.85 | 21.4 | 10.92 |
| **Race** | White, non-Hispanic | 35.82* | 29.98* | 23.84* | 10.37* |
|  | Black, non-Hispanic | 36.68* | 31.92* | 21.03* | 10.37* |
|  | Hispanic | 65.86* | 15.49* | 4.91* | 13.75* |
|  | Other race | 43.67* | 34.72* | 13.96* | 7.65* |
| **Kids** | No kids | 36.66* | 27.83* | 22.76* | 12.75* |
|  | Have kids | 48.1* | 30.23* | 14.86* | 6.81* |
| **Household Size** | 1--2 | 38.17* | 24.71* | 24.17* | 12.96* |
|  | 3--4 | 40.23* | 31.44* | 19.36* | 8.97* |
|  | 5+ | 49.14* | 33.89* | 8.61* | 8.36* |

Highlighted denotes p<0.05

**Table N: Chi-square test: Food waste ensures quality**

| ***FW*^a^ *ensures quality*** | | | | | |
| --- | --- | --- | --- | --- | --- |
|  |  | **Agree Strongly** | **Agree Somewhat** | **Diagree Somewhat** | **Diagree Strongly** |
| **All** |  | 26.91 | 32.38 | 26.52 | 14.2 |
| **Education** | <High School | 42.94* | 26.88* | 20.92* | 9.26* |
|  | High School | 27.66* | 27.59* | 24.68* | 20.07* |
|  | Some College | 25.35* | 29.69* | 30.29* | 14.68* |
|  | Graduate College | 18.68* | 40.6* | 29.81* | 10.92* |
|  | Graduate School | 22.51* | 44.38* | 24.63* | 8.48* |
| **Household Income** | Refused/Missing | 23.47* | 35.89* | 24.06* | 16.58* |
|  | Less than $50,000 | 32.91* | 27.94* | 25.27* | 13.89* |
|  | $50,000-$99,999 | 17.11* | 31.31* | 35.79* | 15.79* |
|  | More than $100,000 | 22.47* | 47.18* | 18.66* | 11.69* |
| **Age** | 18-29 | 26.89* | 24.78* | 30.15* | 18.18* |
|  | 30-49 | 20.28* | 41.25* | 26.49* | 11.98* |
|  | 50-64 | 34.87* | 28.3* | 26.16* | 10.67* |
|  | 65+ | 28.33* | 30.16* | 23.2* | 18.32* |
| **Gender** | Male | 25.08 | 31.5 | 24.88 | 18.54 |
|  | Female | 28.61 | 33.19 | 28.04 | 10.15 |
| **Race** | White, non-Hispanic | 24.25 | 32.43 | 29.55 | 13.77 |
|  | Black, non-Hispanic | 27.09 | 29.64 | 22.88 | 20.4 |
|  | Hispanic | 40.74 | 25.78 | 18.86 | 14.62 |
|  | Other race | 26.74 | 42.81 | 21.45 | 9 |
| **Kids** | No kids | 27.07 | 30.41 | 29.27 | 13.25 |
|  | Have kids | 26.65 | 35.54 | 22.09 | 15.72 |
| **Household Size** | 1--2 | 28.08 | 31.62 | 28.01 | 12.3 |
|  | 3--4 | 24.77 | 33.23 | 27.11 | 14.9 |
|  | 5+ | 30.67 | 28.55 | 23.33 | 17.45 |

Highlighted denotes p<0.05

**Table O: Chi-square test: Food waste is bad for environment**

| ***FW*^a^ *bad for environment*** | | | | | |
| --- | --- | --- | --- | --- | --- |
|  |  | **Agree Strongly** | **Agree Somewhat** | **Diagree Somewhat** | **Diagree Strongly** |
| **All** |  | 35.64 | 22.75 | 28.89 | 12.72 |
| **Education** | <High School | 39.2* | 15.53* | 19.68* | 25.59* |
|  | High School | 30.39* | 21.41* | 35.41* | 12.79* |
|  | Some College | 36.36* | 19.31* | 36.87* | 7.47* |
|  | Graduate College | 26.31* | 40.26* | 21.6* | 11.83* |
|  | Graduate School | 54.52* | 19.15* | 14.91* | 11.43* |
| **Household Income** | Refused/Missing | 44.61 | 15.33 | 25.6 | 14.46 |
|  | Less than $50,000 | 38.09 | 19.49 | 29.27 | 13.15 |
|  | $50,000-$99,999 | 34.27 | 26.18 | 30.47 | 9.08 |
|  | More than $100,000 | 24.71 | 32.71 | 27.11 | 15.47 |
| **Age** | 18-29 | 45.88* | 19.84* | 28.09* | 6.18* |
|  | 30-49 | 29.96* | 25.28* | 32.55* | 12.21* |
|  | 50-64 | 36.32* | 23.41* | 28.18* | 12.08* |
|  | 65+ | 32.51* | 20.71* | 24.16* | 22.62* |
| **Gender** | Male | 34.29* | 26.33* | 21.86* | 17.52* |
|  | Female | 36.92* | 19.33* | 35.61* | 8.14* |
| **Race** | White, non-Hispanic | 32.92 | 24.5 | 28.92 | 13.67 |
|  | Black, non-Hispanic | 28.84 | 21.07 | 33.17 | 16.91 |
|  | Hispanic | 44.9 | 19.9 | 24.18 | 11.03 |
|  | Other race | 47.7 | 19.41 | 29 | 3.9 |
| **Kids** | No kids | 37.76 | 21.53 | 27.65 | 13.06 |
|  | Have kids | 32.23 | 24.71 | 30.88 | 12.17 |
| **Household Size** | 1--2 | 36.43* | 22.74* | 26.1* | 14.73* |
|  | 3--4 | 36.41* | 16.8* | 34.77* | 12.02* |
|  | 5+ | 32.4* | 37.81* | 19.93* | 9.86* |

Highlighted denotes p<0.05

**Table P: Chi-square test: More food is wasted when buy in bulk**

| ***More FW*^a^ *when buy in bulk*** | | | | | |
| --- | --- | --- | --- | --- | --- |
|  |  | **Agree Strongly** | **Agree Somewhat** | **Diagree Somewhat** | **Diagree Strongly** |
| **All** |  | 25.64 | 27.24 | 22.46 | 24.66 |
| **Education** | <High School | 19.72 | 17.72 | 27.43 | 35.13 |
|  | High School | 30.56 | 21.92 | 23.87 | 23.65 |
|  | Some College | 26.52 | 29.31 | 18.66 | 25.51 |
|  | Graduate College | 20.8 | 29.91 | 24.09 | 25.2 |
|  | Graduate School | 23.16 | 40.53 | 21.16 | 15.16 |
| **Household Income** | Refused/Missing | 15.07 | 26.19 | 16.18 | 42.57 |
|  | Less than $50,000 | 30.43 | 22.39 | 21.11 | 26.08 |
|  | $50,000-$99,999 | 21.61 | 32.22 | 25.03 | 21.14 |
|  | More than $100,000 | 21 | 36.93 | 26.68 | 15.39 |
| **Age** | 18-29 | 29.41 | 30 | 16.49 | 24.1 |
|  | 30-49 | 22.86 | 32.08 | 26.38 | 18.69 |
|  | 50-64 | 25.28 | 23.82 | 23.88 | 27.02 |
|  | 65+ | 27.02 | 19.88 | 19.95 | 33.16 |
| **Gender** | Male | 23.93 | 30.01 | 21.96 | 24.09 |
|  | Female | 27.23 | 24.67 | 22.92 | 25.18 |
| **Race** | White, non-Hispanic | 22.41* | 26.89* | 25.46* | 25.24* |
|  | Black, non-Hispanic | 26.63* | 25.9* | 18.99* | 28.47* |
|  | Hispanic | 28.8* | 29.25* | 17.07* | 24.88* |
|  | Other race | 49.58* | 25.35* | 10.43* | 14.64* |
| **Kids** | No kids | 26.6 | 25.07 | 22.4 | 25.93 |
|  | Have kids | 24.07 | 30.81 | 22.56 | 22.57 |
| **Household Size** | 1--2 | 25.02* | 28.08* | 20.16* | 26.73* |
|  | 3--4 | 19.87* | 32.35* | 27.86* | 19.92* |
|  | 5+ | 43.2* | 12.41* | 16.86* | 27.53* |

Highlighted denotes p<0.05

**Table Q: Chi-square test: Hard to reduce food waste further**

| ***Hard to reduce FW*^a^ *further*** | | | | | |
| --- | --- | --- | --- | --- | --- |
|  |  | **Agree Strongly** | **Agree Somewhat** | **Diagree Somewhat** | **Diagree Strongly** |
| **All** |  | 32.87 | 18.31 | 27.76 | 21.06 |
| **Education** | <High School | 36.1 | 12.61 | 29.1 | 22.18 |
|  | High School | 39.97 | 16.22 | 24.06 | 19.74 |
|  | Some College | 27.63 | 18.27 | 30.75 | 23.35 |
|  | Graduate College | 27.47 | 26.69 | 30.56 | 15.28 |
|  | Graduate School | 30.17 | 18.55 | 25.8 | 25.48 |
| **Household Income** | Refused/Missing | 40.42* | 15.76* | 38.86* | 4.96* |
|  | Less than $50,000 | 37.7* | 14.52* | 25.98* | 21.8* |
|  | $50,000-$99,999 | 23.19* | 20.69* | 27.91* | 28.22* |
|  | More than $100,000 | 26.27* | 29.22* | 28.01* | 16.49* |
| **Age** | 18-29 | 24.13* | 23.83* | 34.47* | 17.57* |
|  | 30-49 | 29.44* | 21.18* | 29.33* | 20.05* |
|  | 50-64 | 32.75* | 14.64* | 27.89* | 24.72* |
|  | 65+ | 49.25* | 11.38* | 17.35* | 22.02* |
| **Gender** | Male | 32.24 | 20.93 | 23.08 | 23.75 |
|  | Female | 33.46 | 15.87 | 32.12 | 18.55 |
| **Race** | White, non-Hispanic | 31.78 | 20.1 | 29.46 | 18.66 |
|  | Black, non-Hispanic | 24.94 | 21.73 | 29.77 | 23.57 |
|  | Hispanic | 39.67 | 13.02 | 20.54 | 26.78 |
|  | Other race | 40.97 | 9.82 | 19.75 | 29.46 |
| **Kids** | No kids | 35.29 | 18.93 | 24.18 | 21.59 |
|  | Have kids | 28.99 | 17.32 | 33.5 | 20.19 |
| **Household Size** | 1--2 | 38.51* | 19.95* | 21.38* | 20.17* |
|  | 3--4 | 23.26* | 16.73* | 34.61* | 25.4* |
|  | 5+ | 37.83* | 16.97* | 31.88* | 13.31* |

Highlighted denotes p<0.05

**Table R: Chi-square test: Food waste is a waste of money**

| ***FW*^a^ *is a waste of money*** | | | | | |
| --- | --- | --- | --- | --- | --- |
|  |  | **Agree Strongly** | **Agree Somewhat** | **Diagree Somewhat** | **Diagree Strongly** |
| **All** |  | 25.47 | 16.61 | 21.19 | 36.74 |
| **Education** | <High School | 31.9 | 9.08 | 12 | 47.02 |
|  | High School | 29.37 | 14.75 | 21.87 | 34.01 |
|  | Some College | 24.26 | 16.92 | 24.34 | 34.48 |
|  | Graduate College | 18.12 | 27.48 | 20.06 | 34.34 |
|  | Graduate School | 21.04 | 14.1 | 22.93 | 41.92 |
| **Household Income** | Refused/Missing | 23.75* | 6.57* | 21.96* | 47.72* |
|  | Less than $50,000 | 28.63* | 17.16* | 18.53* | 35.68* |
|  | $50,000-$99,999 | 26.57* | 10.03* | 27.1* | 36.3* |
|  | More than $100,000 | 14.03* | 29.42* | 21.39* | 35.16* |
| **Age** | 18-29 | 20.54* | 21.75* | 27.86* | 29.86* |
|  | 30-49 | 30.28* | 17.65* | 15.79* | 36.29* |
|  | 50-64 | 27.53* | 14.13* | 24.22* | 34.12* |
|  | 65+ | 19.46* | 12.29* | 19.38* | 48.87* |
| **Gender** | Male | 26 | 15.52 | 21.16 | 37.32 |
|  | Female | 24.96 | 17.63 | 21.21 | 36.2 |
| **Race** | White, non-Hispanic | 20.99* | 16.35* | 23.39* | 39.27* |
|  | Black, non-Hispanic | 35.26* | 17.2* | 20.22* | 27.32* |
|  | Hispanic | 32.08* | 9.24* | 20.74* | 37.93* |
|  | Other race | 35.01* | 28.21* | 4.3* | 32.48* |
| **Kids** | No kids | 20.74* | 16.23* | 22.01* | 41.03* |
|  | Have kids | 33.06* | 17.22* | 19.86* | 29.86* |
| **Household Size** | 1--2 | 20.84* | 17.43* | 21.2* | 40.52* |
|  | 3--4 | 26.79* | 13.3* | 24.58* | 35.34* |
|  | 5+ | 37.67* | 22.2* | 14.92* | 25.21* |

Highlighted denotes p<0.05

**Table S: Chi-square test: No time to worry about food waste**

| ***No time to worry about FW*^a^** | | | | | |
| --- | --- | --- | --- | --- | --- |
|  |  | **Agree Strongly** | **Agree Somewhat** | **Diagree Somewhat** | **Diagree Strongly** |
| **All** |  | 10.6 | 13.52 | 28.04 | 47.84 |
| **Education** | <High School | 14.4 | 17.84 | 28.85 | 38.91 |
|  | High School | 12.57 | 15.31 | 22.82 | 49.31 |
|  | Some College | 8.98 | 13.67 | 25.08 | 52.27 |
|  | Graduate College | 8.12 | 7.91 | 40.72 | 43.25 |
|  | Graduate School | 7.77 | 12.25 | 30.58 | 49.4 |
| **Household Income** | Refused/Missing | 4.85 | 20.24 | 25.43 | 49.49 |
|  | Less than $50,000 | 12.33 | 12.73 | 24.14 | 50.8 |
|  | $50,000-$99,999 | 5.61 | 14.25 | 32.21 | 47.93 |
|  | More than $100,000 | 14.65 | 11.81 | 36.65 | 36.89 |
| **Age** | 18-29 | 3.08* | 18.34* | 34.92* | 43.65* |
|  | 30-49 | 9.5* | 13.17* | 33.77* | 43.56* |
|  | 50-64 | 16.59* | 13.46* | 20.71* | 49.24* |
|  | 65+ | 13.22* | 8.63* | 18.71* | 59.44* |
| **Gender** | Male | 10.8 | 14.62 | 27.53 | 47.05 |
|  | Female | 10.42 | 12.49 | 28.51 | 48.59 |
| **Race** | White, non-Hispanic | 8.71* | 13.94* | 31.16* | 46.19* |
|  | Black, non-Hispanic | 13.59* | 3.24* | 38.44* | 44.72* |
|  | Hispanic | 18.46* | 18.59* | 9.97* | 52.98* |
|  | Other race | 2.87* | 12.92* | 23.91* | 60.3* |
| **Kids** | No kids | 10.85 | 12.04 | 25.17 | 51.94 |
|  | Have kids | 10.2 | 15.93 | 32.71 | 41.16 |
| **Household Size** | 1--2 | 9.49* | 11.7* | 24.97* | 53.85* |
|  | 3--4 | 10.23* | 10.6* | 30.25* | 48.92* |
|  | 5+ | 15.68* | 25.47* | 33.46* | 25.39* |

Highlighted denotes p<0.05

**Table T: Chi-square test: Waste more food than others**

| ***More FW*^a^ *than others*** | | | | | |
| --- | --- | --- | --- | --- | --- |
|  |  | **Agree Strongly** | **Agree Somewhat** | **Diagree Somewhat** | **Diagree Strongly** |
| **All** |  | 5.58 | 7.96 | 26.8 | 59.66 |
| **Education** | <High School | 11.98* | 12.63* | 16.1* | 59.28* |
|  | High School | 5.95* | 7.44* | 20.91* | 65.7* |
|  | Some College | 2.97* | 11.44* | 29.55* | 56.04* |
|  | Graduate College | 3.81* | 3.51* | 36.31* | 56.37* |
|  | Graduate School | 5.04* | 3.05* | 33.59* | 58.32* |
| **Household Income** | Refused/Missing | 1.28* | 8.79* | 17.62* | 72.31* |
|  | Less than $50,000 | 7.65* | 9.23* | 19.87* | 63.26* |
|  | $50,000-$99,999 | 3.12* | 3.4* | 40.66* | 52.82* |
|  | More than $100,000 | 3.94* | 9.56* | 36.29* | 50.21* |
| **Age** | 18-29 | 4.75* | 13.11* | 31.59* | 50.54* |
|  | 30-49 | 5.05* | 5.17* | 31.75* | 58.03* |
|  | 50-64 | 9.03* | 9.75* | 21.45* | 59.76* |
|  | 65+ | 2.72* | 4.44* | 19.23* | 73.62* |
| **Gender** | Male | 5.69 | 9.53 | 23.01 | 61.77 |
|  | Female | 5.48 | 6.56 | 30.18 | 57.77 |
| **Race** | White, non-Hispanic | 4.23* | 5.81* | 33.45* | 56.51* |
|  | Black, non-Hispanic | 4.41* | 8.64* | 22.67* | 64.28* |
|  | Hispanic | 9.73* | 15.7* | 8.81* | 65.76* |
|  | Other race | 11.93* | 5.33* | 13.59* | 69.15* |
| **Kids** | No kids | 5.88* | 5.55* | 23.56* | 65.01* |
|  | Have kids | 5.08* | 11.91* | 32.07* | 50.93* |
| **Household Size** | 1--2 | 4.58* | 5.94* | 23.03* | 66.46* |
|  | 3--4 | 7.33* | 5.14* | 31.3* | 56.23* |
|  | 5+ | 4.8* | 19.38* | 29.27* | 46.55* |

Highlighted denotes p<0.05

a - FW – food waste

b - FB – foodborne.
