# Supplementary material for: Household Food Waste: Multivariate Regression and Principal Components Analyses of Awareness and Attitudes among U.S. Consumers
Source: PLoS One. 2016 Jul 21;11(7):e0159250. doi: 10.1371/journal.pone.0159250 (PMC4956302; doi:10.1371/journal.pone.0159250)
Supplement: S2 File — (DOC) [file pone.0159250.s002.doc]

**Supporting Information: Questionnaire**

Q METRO

R 1 1027^1 --> 27=1 Center City (Metro)

R 2 1027^2 --> 27=2 Center City County (Metro)

R 3 1027^3 --> 27=3 Surburban (Metro)

R 4 1027^4 --> 27=4 Non-Center City (Metro)

R 5 1027^5 --> 27=5 Non-Metro

R 0 1027^0 --> 27=0 No metro status(refused)

Q REGION

R 1 1029^1 --> 28=1 North East

R 2 1029^2 --> 28=2 North Central

R 3 1029^3 --> 28=3 South

R 4 1029^4 --> 28=4 West

Q OWNHOME

T

T Z-1. Is your home owned or rented?

T

R 1 1066^1 --> 30=1 Owned

R 2 1066^2 --> 30=2 Rented

R 8 1066^8 --> 30=8 Don't know

R 9 1066^9 --> 30=9 Refused

Q MSTATUS

T Z-2. Are you ...?

T

R 1 1067^1 --> 31=1 Single, that is never married

R 2 1067^2 --> 31=2 Single, living with a partner

R 3 1067^3 --> 31=3 Married

R 4 1067^4 --> 31=4 Separated

R 5 1067^5 --> 31=5 Widowed

R 6 1067^6 --> 31=6 Divorced

R 9 1067^9 --> 31=9 Refused

Q EMPLY

T

T Employment Status

T

R 01 1923^1 --> 32.2=01 Full-time

R 02 1923^2 --> 32.2=02 Part-time

R 03 1923^3 --> 32.2=03 Retired

R 04 1923^4 --> 32.2=04 Homemaker

R 05 1923^5 --> 32.2=05 Student

R 06 1923^6 --> 32.2=06 Temporarily unemployed

R 07 1923^7 --> 32.2=07 Disabled/Handicapped

R 08 1923^8 --> 32.2=08 Other not employed

R 09 1923^9 --> 32.2=09 Refused

Q HHCOMP

T Z-6. Including yourself, how many people are there living in your household?

R 1 1072^1 --> 34=1 One

R 2 1072^2 --> 34=2 Two

R 3 1072^3 --> 34=3 Three

R 4 1072^4 --> 34=4 Four

R 5 1072^5 --> 34=5 Five

R 6 1072^6 --> 34=6 Six

R 7 1072^7 --> 34=7 Seven

R 8 1072^8 --> 34=8 Eight or more

R 9 1072^9 --> 34=9 Refused

Q ADULTF

T

T Z-6Ab. How many of these adults are female?

T

R 1 1078^1 --> 37=1 One

R 2 1078^2 --> 37=2 Two

R 3 1078^3 --> 37=3 Three

R 4 1078^4 --> 37=4 Four

R 5 1078^5 --> 37=5 Five

R 6 1078^6 --> 37=6 Six

R 7 1078^7 --> 37=7 Seven

R 8 1078^8 --> 37=8 Eight or more

R 0 1078^0 --> 37=0 None

R 9 1078^9 --> 37=9 Refused

Q KIDS1217

T

T Z-6B. How many are children 12 to 17?

T

T

R 0 1074^0 --> 38=0 None

R 1 1074^1 --> 38=1 One

R 2 1074^2 --> 38=2 Two

R 3 1074^3 --> 38=3 Three

R 4 1074^4 --> 38=4 Four

R 5 1074^5 --> 38=5 Five

R 6 1074^6 --> 38=6 Six

R 7 1074^7 --> 38=7 Seven

R 8 1074^8 --> 38=8 Eight or more

R 9 1074^9 --> 38=9 Refused

Q KIDS611

T Z-6C. How many are children 6 to 11?

T

R 0 1075^0 --> 39=0 None

R 1 1075^1 --> 39=1 One

R 2 1075^2 --> 39=2 Two

R 3 1075^3 --> 39=3 Three

R 4 1075^4 --> 39=4 Four

R 5 1075^5 --> 39=5 Five

R 6 1075^6 --> 39=6 Six

R 7 1075^7 --> 39=7 Seven

R 8 1075^8 --> 39=8 Eight or more

R 9 1075^9 --> 39=9 Refused

Q KIDS06

T Z-6D. How many are children under 6?

T

R 0 1076^0 --> 40=0 None

R 1 1076^1 --> 40=1 One

R 2 1076^2 --> 40=2 Two

R 3 1076^3 --> 40=3 Three

R 4 1076^4 --> 40=4 Four

R 5 1076^5 --> 40=5 Five

R 6 1076^6 --> 40=6 Six

R 7 1076^7 --> 40=7 Seven

R 8 1076^8 --> 40=8 Eight or more

R 9 1076^9 --> 40=9 Refused

Q AGE

X Range=18-99 Exceptions=

T

T Z-7. What is your age?

T

Q AGE2

T

T Z-7a. Could you please tell me if you are ...?

T

R 1 1085^1 --> 44=1 18-29

R 2 1085^2 --> 44=2 30-49

R 3 1085^3 --> 44=3 50-64

R 4 1085^4 --> 44=4 65+

R 9 1085^9 --> 44=9 Refused

Q TOTALAGE

T

T reformatted age #1

T

R 1 1929^1 --> 45=1 18-29

R 2 1929^2 --> 45=2 30-49

R 3 1929^3 --> 45=3 50-64

R 4 1929^4 --> 45=4 65+

R 9 1929^9 --> 45=9 Refused

Q EDUC

T

T Z-8. What is the last grade of school you completed?

T

R 1 1083^1 --> 47=1 Less than high school graduate

R 2 1083^2 --> 47=2 High school graduate

R 3 1083^3 --> 47=3 Some college

R 4 1083^4 --> 47=4 Graduated college

R 5 1083^5 --> 47=5 Graduate school or more

R 6 1083^6 --> 47=6 Technical school/other

R 9 1083^9 --> 47=9 Refused

Q INCOME

T Z-9. Is your total annual household income from all sources,

T and before taxes ...?

T

R 01 Less than $15,000

R 02 $15,000 but less than $25,000

R 03 $25,000 but less than $30,000

R 04 $30,000 but less than $40,000

R 05 $40,000 but less than $50,000

R 06 $50,000 but less than $75,000

R 07 $75,000 but less than $100,000

R 08 $100,000 and over (Unspecified)

R 09 Less than $50,000 (Unspecified)

R 10 $50,000 but less than $100,000 (Unspecified)

R 11 Over $100,000

R 12 $100,000 to under $150,000

R 13 $150,000 to under $200,000

R 14 $200,000 to under $250,000

R 15 $250,000 or more

R 98 Don't know

R 99 Refused

Q RACE

T

T Race of Respondent

T

R 01 1925^1 --> 50.2=01 White Non-Hispanic

R 02 1925^2 --> 50.2=02 Black Non-Hispanic

R 03 1925^3 --> 50.2=03 White Hispanic

R 04 1925^4 --> 50.2=04 Black Hispanic

R 05 1925^5 --> 50.2=05 Unspecified Hispanic

R 06 1925^6 --> 50.2=06 Asian/Chinese/Japanese

R 07 1925^7 --> 50.2=07 Native American/American Indian/Alaska Native

R 08 1925^8 --> 50.2=08 Native Hawaiian and other Pacific Islander

R 09 1925^9 --> 50.2=09 Other Race

R 10 1925^X --> 50.2=10 Mixed

R 99 1925^0 --> 50.2=99 Refused

Q AFFILROT

T

T Affiliation rotate

T

R 1 1167^1 --> 52=1 Republican/Democrat

R 2 1167^2 --> 52=2 Democrat/Republican

Q POLAFFIL

T

T Z-11a. Generally speaking, do you usually think of yourself as:

T

R 1 1096^1 --> 53=1 A Republican

R 2 1096^2 --> 53=2 A Democrat

R 3 1096^3 --> 53=3 An Independent

R 0 1096^0 --> 53=0 Other

R 8 1096^8 --> 53=8 Don't know

R 9 1096^9 --> 53=9 Refused

Q SEX

T

T Enter Sex of Respondent

T

R 1 1057^1 --> 58=1 Male

R 2 1057^2 --> 58=2 Female

Q RELI1

T RELIG What is your present religion, if any? Are you Protestant,

T Roman Catholic, Mormon, Orthodox such as Greek or Russian Orthodox, Jewish, Muslim,

T Buddhist, Hindu, atheist, agnostic, something else, or

T nothing in particular?

R 01 6326^1 --> 59.2=01 Baptist

R 20 6327^8 --> 59.2=20 Buddhist

R 02 6326^2 --> 59.2=02 Catholic, Roman Catholic

R 22 6327^0 --> 59.2=22 Christian (Just Christian)

R 26 6328^2 --> 59.2=26 Church of Christ, or Disciples of Christ (Christian Church)

R 27 6328^3 --> 59.2=27 Church of God

R 28 6328^4 --> 59.2=28 Congregational or United Church of Christ (UCC)

R 24 6327^Y --> 59.2=24 Episcopalian or Anglican

R 05 6326^5 --> 59.2=05 Evangelical

R 21 6327^9 --> 59.2=21 Hindu

R 29 6328^5 --> 59.2=29 Holiness (Nazarenes, Wesleyan Church, Salvation Army)

R 06 6326^6 --> 59.2=06 Jehovah's Witness

R 07 6326^7 --> 59.2=07 Jewish/Judaism

R 08 6326^8 --> 59.2=08 Lutheran

R 25 6328^1 --> 59.2=25 Methodist

R 10 6326^0 --> 59.2=10 Mormon (Church of Jesus Christ of Latter-Day Saints/LDS)

R 11 6326^X --> 59.2=11 Muslim/Islamic

R 23 6327^X --> 59.2=23 Non-denominational or Independent Church

R 12 6326^Y --> 59.2=12 Orthodox (Eastern, Greek, Russian, Armenian, etc)

R 13 6327^1 --> 59.2=13 Pentecostal (Assemblies of God, Four-Square Gospel)

R 14 6327^2 --> 59.2=14 Presbyterian

R 15 6327^3 --> 59.2=15 Protestant

R 16 6327^4 --> 59.2=16 Seventh-Day Adventist

R 30 6328^6 --> 59.2=30 Reformed

R 19 6327^7 --> 59.2=19 Unitarian/Universalist

R 95 6345^5 --> 59.2=95 Atheist

R 96 6345^6 --> 59.2=96 Agnostic

R 97 6345^7 --> 59.2=97 Other

R 90 6345^0 --> 59.2=90 Nothing in particular

R 98 6345^8 --> 59.2=98 Don't know

R 99 6345^9 --> 59.2=99 Refused

Q DEMH1

T

T HE-1. Do you have health insurance?

T

R 1 1376^1 --> 74=1 Yes

R 2 1376^2 --> 74=2 No

R 8 1376^8 --> 74=8 Don't know

R 9 1376^9 --> 74=9 Refused

Q FOOD1

T

T FW-1. We would now like to ask some questions concerning the food you buy,

T prepare and eat in your home. Your participation in this section of questions is

T voluntary and you may skip any questions in this block for any reason. Are you

T willing to answer these questions?

R 1 6016^1 --> 79=1 Yes

R 2 6016^2 --> 79=2 No [skip section]

R 3 6016^3 --> 79=3 Uncertain [skip section]

R 9 6016^9 --> 79=9 Refused [skip section]

Q FOOD2

T

T FW-2. In the last 12 months, have you read, seen or heard anything about

T the amount of food that is wasted or about ways to reduce the amount

T of food that is wasted?

T

R 1 6017^1 --> 80=1 Yes

R 2 6017^2 --> 80=2 No

R 3 6017^3 --> 80=3 Uncertain

R 9 6017^9 --> 80=9 Refused

Q FOOD3ROT

R A

R B

R C

R D

R E

R F

R G

R H

R I

Q FOOD3A

T

T

T FW-3a. To what extent would you agree with the following statements about food

T that is served in your home that gets thrown away?

T * Throwing away food is bad for the environment*

T

R 1 6037^1 --> 90=1 Agree strongly

R 2 6037^2 --> 90=2 Agree somewhat

R 3 6037^3 --> 90=3 Disagree somewhat

R 4 6037^4 --> 90=4 Disagree strongly

R 8 6037^8 --> 90=8 Don't Know

R 9 6037^9 --> 90=9 Refused

Q FOOD3B

T

T

T FW-3b. To what extent would you agree with the following statements about food

T that is served in your home that gets thrown away?

T * Throwing away food is a major source of wasted money in your household *

T

R 1 6038^1 --> 91=1 Agree strongly

R 2 6038^2 --> 91=2 Agree somewhat

R 3 6038^3 --> 91=3 Disagree somewhat

R 4 6038^4 --> 91=4 Disagree strongly

R 8 6038^8 --> 91=8 Don't Know

R 9 6038^9 --> 91=9 Refused

Q FOOD3C

T

T FW-3c. To what extent would you agree with the following statements about food

T that is served in your home that gets thrown away?

T * Throwing away food if the package date has passed reduces the chance someone will get sick from eating the food *

T

R 1 6039^1 --> 92=1 Agree strongly

R 2 6039^2 --> 92=2 Agree somewhat

R 3 6039^3 --> 92=3 Disagree somewhat

R 4 6039^4 --> 92=4 Disagree strongly

R 8 6039^8 --> 92=8 Don't Know

R 9 6039^9 --> 92=9 Refused

Q FOOD3D

T

T FW-3d. To what extent would you agree with the following statements about food

T that is served in your home that gets thrown away?

T * You feel guilty when you throw away food *

R 1 6040^1 --> 93=1 Agree strongly

R 2 6040^2 --> 93=2 Agree somewhat

R 3 6040^3 --> 93=3 Disagree somewhat

R 4 6040^4 --> 93=4 Disagree strongly

R 8 6040^8 --> 93=8 Don't Know

R 9 6040^9 --> 93=9 Refused

Q FOOD3E

T

T FW-3e. To what extent would you agree with the following statements about food

T that is served in your home that gets thrown away?

T * You don't have enough time to worry about the amount of food you waste *

T

R 1 6041^1 --> 94=1 Agree strongly

R 2 6041^2 --> 94=2 Agree somewhat

R 3 6041^3 --> 94=3 Disagree somewhat

R 4 6041^4 --> 94=4 Disagree strongly

R 8 6041^8 --> 94=8 Don't Know

R 9 6041^9 --> 94=9 Refused

Q FOOD3F

T

T FW-3f. To what extent would you agree with the following statements about food

T that is served in your home that gets thrown away?

T * Some food waste is necessary to make sure meals taste fresh and good *

T

R 1 6042^1 --> 95=1 Agree strongly

R 2 6042^2 --> 95=2 Agree somewhat

R 3 6042^3 --> 95=3 Disagree somewhat

R 4 6042^4 --> 95=4 Disagree strongly

R 8 6042^8 --> 95=8 Don't Know

R 9 6042^9 --> 95=9 Refused

Q FOOD3G

T

T FW-3g. To what extent would you agree with the following statements about food

T that is served in your home that gets thrown away?

T * It would be difficult to reduce your household's food waste any further *

R 1 6043^1 --> 96=1 Agree strongly

R 2 6043^2 --> 96=2 Agree somewhat

R 3 6043^3 --> 96=3 Disagree somewhat

R 4 6043^4 --> 96=4 Disagree strongly

R 8 6043^8 --> 96=8 Don't Know

R 9 6043^9 --> 96=9 Refused

Q FOOD3H

T

T FW-3h. To what extent would you agree with the following statements about food

T that is served in your home that gets thrown away?

T * You waste more food when you buy things in large packages or when you buy in large quantities during a sale *

T

R 1 6044^1 --> 97=1 Agree strongly

R 2 6044^2 --> 97=2 Agree somewhat

R 3 6044^3 --> 97=3 Disagree somewhat

R 4 6044^4 --> 97=4 Disagree strongly

R 8 6044^8 --> 97=8 Don't Know

R 9 6044^9 --> 97=9 Refused

Q FOOD3I

T

T FW-3i. To what extent would you agree with the following statements about food

T that is served in your home that gets thrown away?

T * Your household wastes more food than other households of your size *

T

R 1 6045^1 --> 98=1 Agree strongly

R 2 6045^2 --> 98=2 Agree somewhat

R 3 6045^3 --> 98=3 Disagree somewhat

R 4 6045^4 --> 98=4 Disagree strongly

R 8 6045^8 --> 98=8 Don't Know

R 9 6045^9 --> 98=9 Refused
